# Comparison of the safety profiles for pirfenidone and nintedanib: a disproportionality analysis of the US food and drug administration adverse event reporting system

**DOI:** 10.3389/fphar.2024.1256649

**Published:** 2024-05-27

**Authors:** Xiangyu Sun, Huaguang Wang, Xi Zhan, Yuanyuan Yan, Kun Chen, Zhuoling An, Hong Zhou

**Affiliations:** ^1^ Pharmacy Department of Beijing Chao-Yang Hospital, Capital Medical University, Beijing, China; ^2^ Medicines and Equipment Department, Beijing Chaoyang Emergency Medical Rescuing Center, Beijing, China; ^3^ Department of Critical Care and Pulmonary Medicine, Beijing Chaoyang Hospital, Capital Medical University, Beijing, China; ^4^ Pharmacy Department of Aviation General Hospital, Beijing, China; ^5^ Beijing Chaoyang Emergency Medical Rescuing Center, Beijing, China

**Keywords:** idiopathic pulmonary fibrosis, pirfenidone, nintedanib, FAERS, OpenVigil 2.1, adverse events, designated medical events

## Abstract

**Background:**

Idiopathic pulmonary fibrosis (IPF) is a chronic, progressive interstitial lung disease of unknown etiology. Pirfenidone (PFD) and nintedanib (NDN) were both conditionally recommended in the clinical practice guideline published in 2015. Safety and tolerability are related to the risk of treatment discontinuation. Therefore, this study evaluated and compared the adverse events (AEs) of PFD and NDN in a large real-world population by analyzing data from the FDA Adverse Event Reporting System (FAERS) to provide a reference for their rational and safe use.

**Methods:**

The AEs of PFD and NDN were extracted from the FAERS database. The pharmacovigilance online analysis tool OpenVigil 2.1 was used to retrieve data from the FAERS database from the first quarter of 2012 to the second quarter of 2022. The reporting odds ratio (ROR) and proportional reporting ratio were used to detect the risk signals.

**Results:**

The database included 26,728 and 11,720 reports for PFD and NDN, respectively. The most frequent AEs of PFD and NDN were gastrointestinal disorders. The RORs for these drugs were 5.874 and 5.899, respectively. “Cardiac disorders” was the most statistically significant system order class for NDN with an ROR of 9.382 (95% confidence interval = 8.308–10.594). Furthermore, the numbers of designated medical events of PFD and NDN were 552 and 656, respectively. Notably, liver injury was reported more frequently for NDN (11.096%) than for PFD (6.076%).

**Conclusion:**

This study revealed differences in the reporting of AEs between PFD and NDN. The findings provide reference for physicians in clinical practice. Attention should be paid to the risks of cardiac disorders and liver injury associated with NDN.

## Introduction

Idiopathic pulmonary fibrosis (IPF) is a chronic, progressive interstitial lung disease of unknown etiology (Spagnolo et al., 2021a). It primarily occurs in older men, and its prognosis is poor, with a median survival of only 2–5 years (Raghu et al., 2011; Jo et al., 2017). Globally, the incidence of IPF is increasing. A recent analysis of 22 studies covering 12 countries found that the adjusted incidence and prevalence of IPF range 0.09–1.30 and 0.33–4.51 per 10,000 persons, respectively (Maher et al., 2021). At present, lung transplantation is considered the only effective intervention to improve the life expectancy of patients with IPF, and it has the advantage of improving both symptoms and survival time (Glass et al., 2022). However, because of the limited number of organ donors and patients’ economic status, age, and comorbidities, only a few patients can undergo this treatment (Glass et al., 2020).

In 2015, an updated guideline of the 2011 version was released by the American Thoracic Society (ATS), European Respiratory Society (ERS), Japanese Respiratory Society (JRS), and Latin America Thoracic Association (ALAT) to focus on treatment interventions. Pirfenidone (PFD, Esbriet) and nintedanib (NDN, Ofev) were both conditionally recommended in this guideline, meaning that physicians should choose different treatment therapies consistent with patients’ values and preferences (Raghu et al., 2011). PFD (5-methyl-1-phenyl-2-[1H]-pyridone) is a novel anti-fibrotic and anti-inflammatory drug that can alleviate the deterioration of pulmonary function in patients with IPF (Noble et al., 2011). It was approved for the treatment of adults with mild-to-moderate IPF in the European Union in 2011 and for the treatment of IPF in the United States in 2014 (Spagnolo et al., 2021b). NDN is a multi-target tyrosine kinase inhibitor that can simultaneously block the fibroblast growth factor, vascular endothelial growth factor, and platelet-derived growth factor receptors (Lamb, 2021). The TOMORROW trial, a 52-week multi-country, double-blind, randomized, placebo-controlled phase II clinical trial, proved that NDN reduces the decline in forced vital capacity associated with IPF (Richeldi et al., 2011). The FDA and EMA approved NDN for the treatment of IPF in 2014 and 2015, respectively (Robalo-Cordeiro et al., 2017).

In November 2020, the UK Medicines and Health Products Regulatory Agency (MHRA, 2020) issued an information warning that PFD carries a risk of serious liver injury and updated advice on liver function testing. The Japanese Pharmaceuticals and Medical Devices Agency published pharmaceuticals safety information on NDN detailing cases of thrombocytopenia, including serious cases leading to bleeding and potentially leading nephrotic syndrome in August 2016 and April 2022 (PMDA, 2016; PMDA, 2022). On 14 December 2021, the Australian Government Department of Health and Age Care updated the product information of NDN to include new warnings on ischemic colitis and renal impairment (Medicines Safety Update, 2022).

Although physicians pay more attention to adverse drug reaction warnings regarding PFD and NDN, most warnings were reported in clinical trials or case reports. There is a lack of systematic and comprehensive pharmacovigilance studies regarding these two drugs. Therefore, we evaluated and compared PFD and NDN in a large real-world population by analyzing adverse events (AEs) reported to the FDA Adverse Event Reporting System (FAERS).

## Materials and methods

### Data source

The data of this study were obtained from the pharmacovigilance database of FAERS, which was freely released to the public. The data structure of FAERS adhered to the international safety reporting guidance issued by the International Conference on Harmonisation (ICH E2B) (Neha et al., 2020). However, FAERS data are incomplete because of insufficient and excessive reporting, a lack of denominators, data errors, and duplication (Wang et al., 2022). Therefore, we used OpenVigil 2.1, a freely available pharmacovigilance tool based on the external drug databases Drugbank (https://www.drugbank.ca/) and drugs@FDA (https://www.accessdata.fda.gov/scripts/cder/daf/index.cfm), to map the information in the FAERS database. At the same time, only the reports with complete case information were loaded, and thus, case reports were included and subsequently cleaned (Böhm et al., 2016). The classification and standardization of AEs in the FAERS database are coded using the Medical Dictionary for Regulatory Activities.

### Study procedure

The interventions of interest were PFD and NDN. This study included all records in the FAERS database from the first quarter of 2012 to the second quarter of 2022. The DRUG terms searched included “pirfenidone,” “esbriet,” “Pirespa,” “Etary,” “nintedanib,” “Ofev,” “Vargatef,” and “BIBF1120.” Unexpected AEs were defined as any significant AEs that were not listed in the FDA drug labeling (Zhan et al., 2020). The designated medical events (DMEs) (European Medicines Agency, 2020) were selected from the EMA list, which included 62 different reactions.

### Statistical analysis

Disproportionality analysis was performed to analyze signal detection. In pharmacovigilance, the statistical correlation between drugs and AEs was determined via proportional imbalance analysis. The proportional reporting ratio (PRR) and reporting odds ratio (ROR) are frequency statistics that can be interpreted as the relative risk and odds ratio, respectively (Evans et al., 2001; Ooba and Kubota, 2010; Caster et al., 2020; CredibleMeds, 2022). The data and calculation formulas used for PRR and ROR calculation are presented in Table 1. A ROR signal was defined as positive when the number of cases was ≥2 and the lower limit of the 95% confidence interval (CI) was >1. A positive PRR signal was indicated by ≥3 cases, chi-square ≥4, and PRR ≥2. The ROR and PRR data were performed by OpenVigil 2 × 2 contingency table calculator.

**TABLE 1:** Summary of the algorithms used for signal detection.

Algorithms	Equation	Criteria
ROR	ROR = ad/bc	lower limit of 95% CI > 1, a ≥2
	95%CI = e^ln(ROR)±1. 96(1/a+1/b+1/c+1/d)∧0.5^	
PRR	PRR = a(c + d)/c(a + b)	PRR ≥2, χ^2^ ≥ 4, a ≥3
	χ^2^ = [(ad − bc)^2^](a + b + c + d)/[(a + b)(c + d)(a + c)(b + d)]	

SPSS V21.0 software was used for the chi-square test to evaluate the differences between PFD and NDN in demographic data.

## Results

### Descriptive results

The original data were 37702 reports for PFN and 17119 reports for NDN. After data cleaning and de-duplication through the OpenVigil-Data quality and cleansing procedures, 26728 reports of PFD and 11720 reports of NDN were obtained.

**FIGURE 1:**
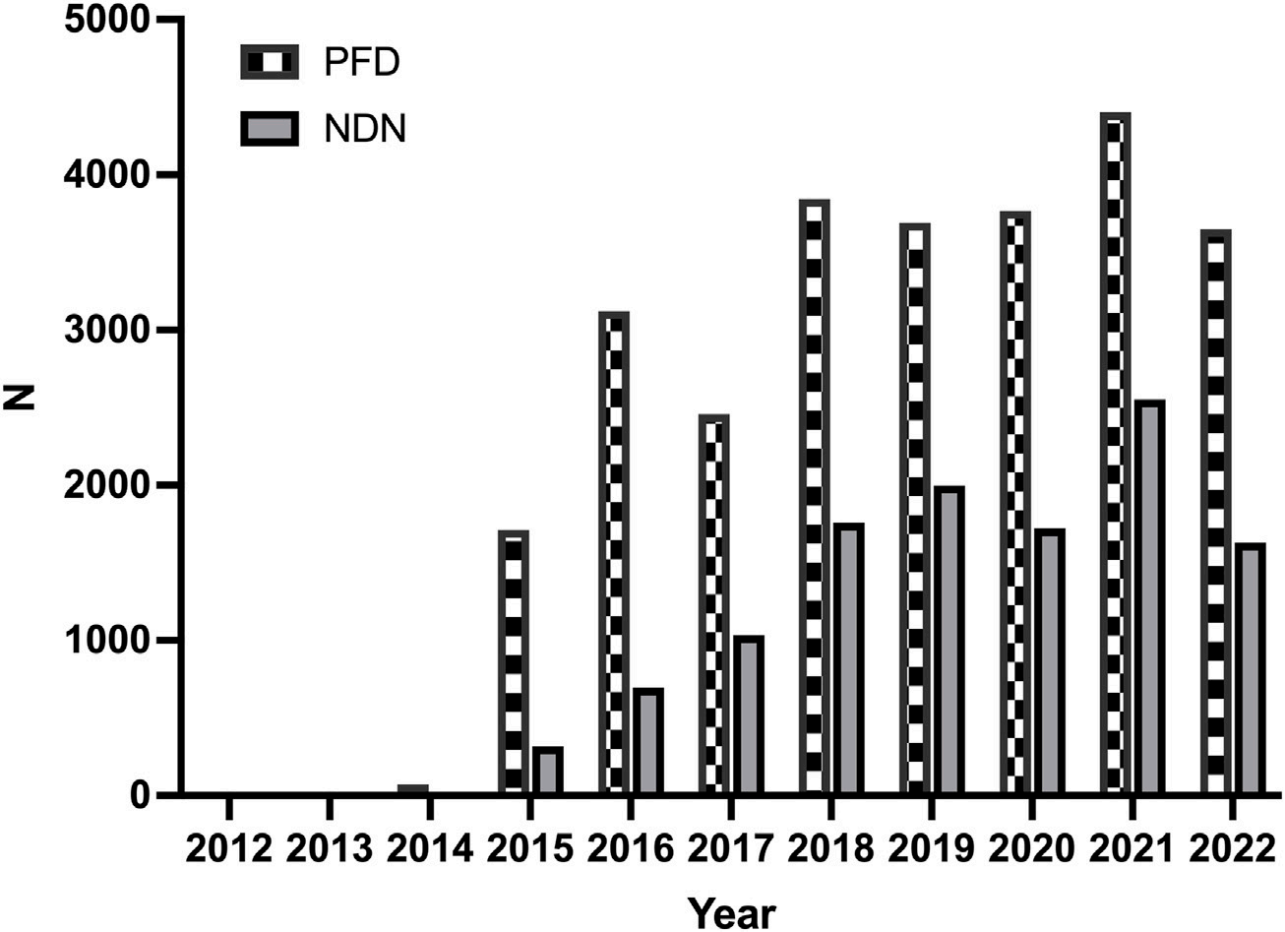
The trends of adverse events reports for pirfenidone and nintedanib from the first quarter of 2012 to the second quarter of 2022.

The trend of the AE reports is presented in Figure 1. The number of reports increased significantly for both two drugs in 2015. The characteristics of the AE reports are presented in Table 2. Events were more common in men, but there was no statistical difference between the two drugs (*p* = 0.703). Although the AE reports for PFD and NDN were concentrated in patients ≥65 years old, in terms of age subgroups, NDN-related AEs more frequently occurred in patients aged 18–65 years than PFD (*p* < 0.0001). Concerning the reporting region, most events were reported in the US for both PFD (86.67%) and NDN (57.10%). Regarding the non-US reports, Great Britain and Japan ranked first for PFD (37.32%) and NDN (22.88%), respectively. For events for which outcome data were available, severe outcomes (including hospitalization, disability, life-threatening, and death) were noted for 73.44% and 72.70% of events related to PFN and NDN, respectively. Among them, death was the most frequently reported severe outcome.

**TABLE 2:** Characteristics of adverse events reported for pirfenidone and nintedanib.

	Pirfenidone n (%)	Nintedanib n (%)	*p*-value
Total number of reports	26728	11720	
Sex			
Number of reports available	25539 (95.55%)	10076 (85.97%)	
Female	9325 (36.51%)	3700 (36.72%)	0.703
Male	16214 (63.49%)	6376 (63.28%)	
Age			
Number of reports available	9048 (33.85%)	6888 (58.77%)	
Age (years), range			
≤18	11 (0.12%)	10 (0.15%)	0.684
18–65	1356 (14.99%)	1586 (23.03%)	**< 0.0001**
≥65	7681 (84.89%)	5292 (76.83%)	**< 0.0001**
Reporter Region			
Number of reports available	26662 (99.75%)	10727 (91.53%)	
US	23109 (86.67%)	6125 (57.10%)	
Non-US	3553 (13.33%)	4602 (42.90%)	
Drug role in event occurrence			
Primary suspect drug/Secondary suspect drug	26180 (97.95%)	10960 (93.52%)	
Concomitant	512 (1.92%)	747 (6.37%)	
Interacting	36 (0.13%)	13 (0.11%)	
Outcome of event			
Number of reports available	12924 (48.42%)	8940 (76.28%)	
Death	5638 (43.62%)	2639 (29.52%)	**< 0.0001**
Hospitalization-initial or prolonged	3697 (28.61%)	3536 (39.55%)	**< 0.0001**
Other events	3429 (26.53%)	2439 (27.28%)	0.219
Life-threatening	103 (0.80%)	237 (2.65%)	**< 0.0001**
Disability	53 (0.41%)	87 (0.97%)	
Required intervention	4 (0.03%)	2 (0.02%)	0.703

Bold type denotes statistical significance (*p* < 0.05).

^a^ Wilcoxon’s rank-sum test.

^b^ Chi-squared test.

### SOCs and PTs

**FIGURE 2:**
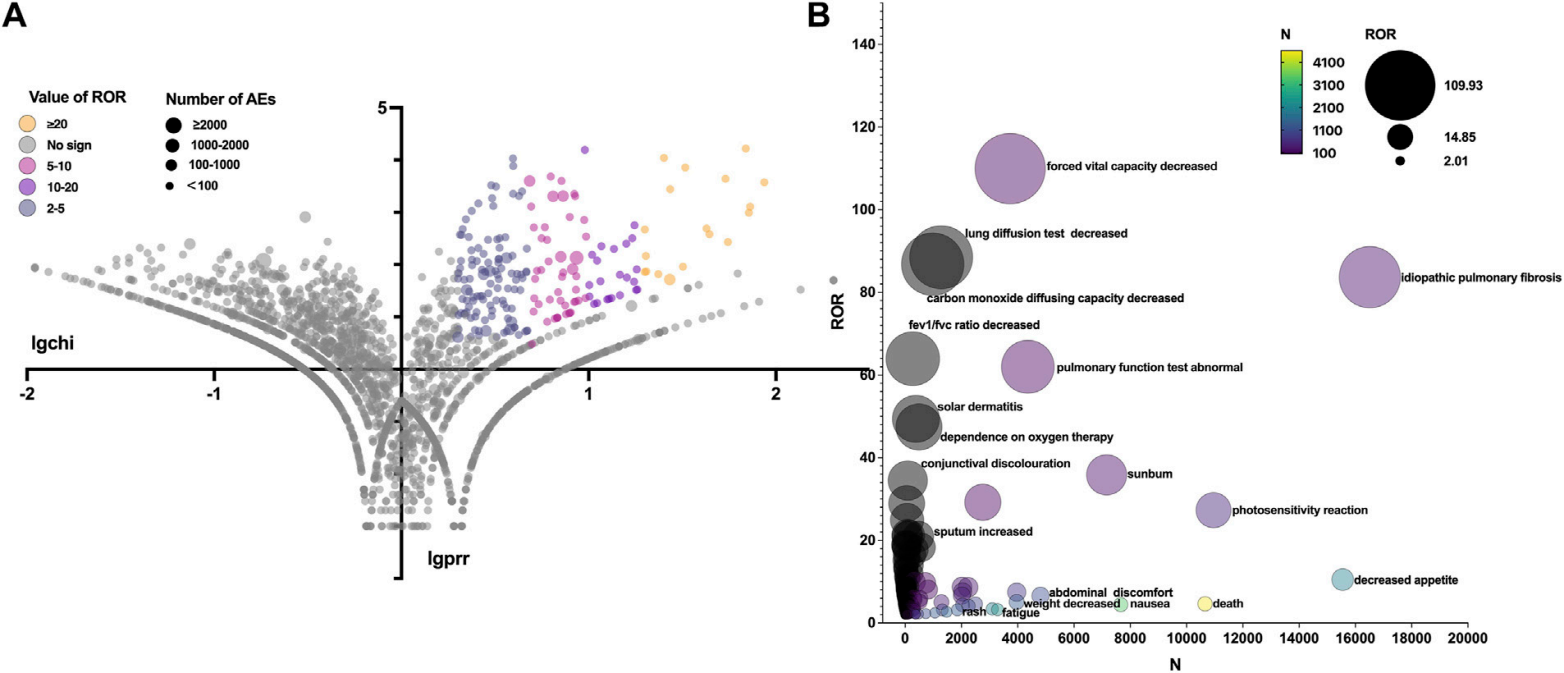
**(A)** Reporting odds ratio (ROR) volcanic map of pirfenidone. The *y*-axis represents the logarithm of the chi-square, and the *x*-axis represents the logarithm of the proportional reporting ratio. In this scatter plot, the points (drugs) in the upper right quadrant had larger signals. **(B)** Bubble chart of preferred terms (PTs).The *x*-axis represents the number of events for PT, and the *y*-axis represents the value of ROR. The size of the bubbles represents the value of ROR, and the color of the bubble represents the number of events per PT.

**FIGURE 3:**
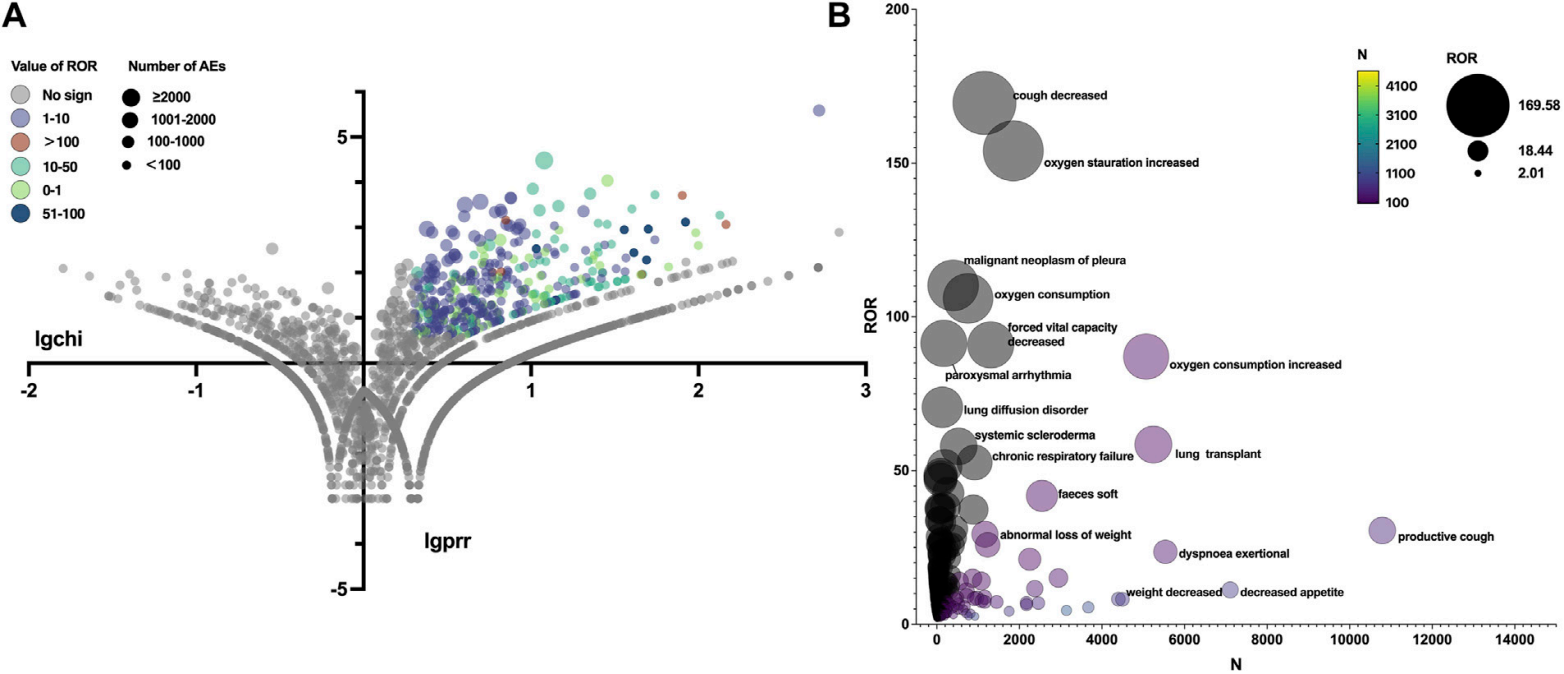
**(A)** Reporting odds ratio (ROR) volcanic map of nintedanib. The *y*-axis represents the logarithm of the chi-square, and the *x*-axis represents the logarithm of the proportional reporting ratio. In this scatter plot, the points (drugs) in the upper right quadrant had larger signals. **(B)** Bubble chart of preferred terms (PTs).The *X*-axis represents the number of AE cases in Nintedanib, and the *Y*-axis represents the ROR value. The size of the bubbles represents the ROR value, and the color of the bubble represents the number of events per PT.

The total AEs of PFD are presented shown in Figure 2A, and the events with significant signals after screening were located in the upper right quadrant. The significant preferred terms (PTs) of interest are presented in Figure 2B and Supplementary Table S2. The top five AEs for PFD were death (4,626, 12.17%), nausea (3,354, 8.82%), fatigue (2,356, 6.20%), decreased appetite (2,016, 5.30%), and diarrhoea (1,999, 5.26%). In addition, unexpected AEs such as aortic aneurysms, amblyopia, and chromaturia were detected in data mining. The top five AEs for NDN were diarrhea (2,933, 11.43%), nausea (1,337, 5.21%), dyspnea (1,130, 4.40%), death (1,097, 4.28%), and decreased appetite (847, 3.30%). The RORs of PFD ranged from 2.006 to 88.407 for 95% CI lower limits exceeding 1. The largest PRR and ROR for forced vital capacity were 109.738 and 109.933, respectively, whereas NDN had the largest PRR and ROR for paroxysmal arrhythmia (91.359 and 91.387, respectively; Supplementary Tables S1, S2). Of note, compared with PFD, the cardiac disorder AEs of NDN should cause concern. In total, 16 PTs were involved, and the most frequently reported terms were atrial fibrillation, cardiac failure, and pericardial effusion (Figure 3; Supplementary Table S2). Some AEs that were listed in the drug labels, such as arthralgia for PFD (N = 275, ROR = 0.719, 95% CI = 0.639–0.810) and back pain (N = 136, ROR = 1.495, 95% CI = 1.262–1.771) for NDN did not have significant signals in our study.

Table 3 summarizes all system organ classes (SOCs) related to PFD and NDN. The most frequently reported SOCs for PFD were “gastrointestinal disorders,” “general disorders and administration site conditions,” and “skin and subcutaneous tissue.” These results were consistent with existing research. However, the most statistically significant SOCs were “eye disorders” (ROR = 11.076, 95% CI = 5.711–21.478), which were not listed in the drug label or reported in any studies. Meanwhile, “gastrointestinal disorders” and “general disorders and administration site conditions” were also reported for NDN, and the other SOC was “respiratory, thoracic, and mediastinal disorders,” which might be related to the progression of the primary disease. Meanwhile, a noteworthy finding was that “cardiac disorders” (ROR = 9.382, 95% CI = 8.308–10.594) was the most statistically significant SOC for NDN.

**TABLE 3:** Frequency of adverse events by system organ class (SOC).

Pirfenidone						Nintedanib							
SOC	N	ROR		*p*-value		SOC			N			ROR	*p*-value
Gastrointestinal disorders	11406	5.874 (5.730–6.022)		<0.001		Gastrointestinal disorders			9249			5.899 (5.780–6.022)	<0.001
General disorders and administration site conditions	9184	3.426 (2.781–4.220)		<0.001		Respiratory, thoracic and mediastinal disorders			5435			6.977 (6.792–7.166)	<0.001
Skin and subcutaneous tissue	3033	3.449 (2.937–3.156)		<0.001		General disorders and administration site conditions			3446			2.699 (2.610–2.792)	<0.001
Respiratory, thoracic, and mediastinal	4692	3.546 (3.932–4.253)		<0.001		Investigations			2045			6.328 (6.058–6.610)	<0.001
Metabolism and nutrition disorders	2199	8.897 (8.443–9.192)		<0.001		Metabolism and nutrition disorders			1317			7.033 (6.660–7.426)	<0.001
Nervous system and psychiatric	1918	2.666 (2.549–2.789)		<0.001		Infections and infestations			1154			4.414 (3.222–3.618)	<0.001
Investigations	2311	5.326 (2.549–2.789)		<0.001		Nervous system disorders			505			3.502 (3.209–3.823)	<0.001
Infections and infestations	1242	3.041 (2.875–3.216)		<0.001		Neoplasms benign, malignant and unspecified (incl cysts and polyps)			500			5.237 (4.796–5.718)	<0.001
Injury, poisoning, and procedural complications	894	7.504 (7.023–8.018)		<0.001		Hepatobiliary disorders			391			3.964 (3.588–4.371)	<0.001
Psychiatric disorders	704	2.163 (2.007–2.330)		<0.001		Vascular disorders			313			2.661 (2.382–2.974)	<0.001
Renal and urinary disorders	91	3.285 (2.672–4.038)		<0.001		Cardiac disorders			263			9.382 (8.308–10.594)	<0.001
Musculoskeletal and connective tissue disorders	65	3.691 (2.891–4.712)		<0.001		Surgical and medical procedures			228			8.393 (7.367–9.562)	<0.001
Surgical and medical procedures	64	7.930 (6.191–10.156)		<0.001		Injury, poisoning and procedural complications			211			2.832 (2.474–3.243)	<0.001
Vascular disorders	62	2.921 (2.275–3.749)		<0.001		Renal and urinary disorders			139			3.628 (3.071–4.286)	<0.001
Social circumstances	40	6.627 (4.485–9.059)		<0.001		Skin and subcutaneous tissue disorders			130			3.095 (2.605–3.676)	<0.001
Neoplasms (benign, malignant, and unspecified)	36	4.324 (3.114–6.006)		<0.001		Blood and lymphatic system disorders			100			3.589 (2.949–4.369)	<0.001
Immune system disorders	33	2.941 (2.088–4.143)		<0.001		Musculoskeletal and connective tissue disorders			93			6.400 (5.219–7.848)	<0.001
Blood and lymphatic system	20	2.184 (1.407–3.390)		<0.001		Immune system disorders			56			7.664 (5.890–9.973)	<0.001
Cardiac and vascular	16	3.969(2.426–6.495)		<0.001		Psychiatric disorders			48			4.504 (3.39–5.98)	<0.001
Eye disorders	9	11.076 (5.711–21.478)		<0.001		Social circumstances			14			2.504 (1.482–4.232)	<0.001
Ear and labyrinth disorders	3	6.673 (2.132–20.886)				Ear and labyrinth disorders			10			7.347 (3.944–13.686)	<0.001
Reproductive system and breast disorders	3	7.991 (2.548–25.054)				Eye disorders			5			4.327 (1.797–10.416)	<0.001
						Reproductive system and breast disorders			3			6.104 (1.962–18.993)	
						Respiratory failure			3			33.773 (10.693–106.671)	

Notes: *p*-values were calculated using the chi-squared test. *p*-values were not calculated for cases with fewer than five events.

### DME results

In total, 552 events covering 32 DMEs were reported for PDF, and 656 events covering 37 DMEs were reported for NDN (Table 4). The patients in the reports for PFD were mainly aged 65–75 years. Meanwhile, the patients in the reports for NDN were most commonly younger than 65 years. DMEs were more common in men than in women for both drugs. NDN was more likely to have serious outcomes (including hospitalization, disability, and death) than PFD. Hearing loss, kidney failure, pancreatitis, blindness, acute kidney injury, and liver failure were reported with high frequencies for PFD. The major DMEs of NDN included febrile neutropenia, acute kidney injury, pancreatitis, kidney failure, and drug-induced liver injury. Notably, liver damage was reported more frequently for NDN (11.096%) than for PFD (6.076%).

**TABLE 4:** The designated medical events for pirfenidone and nintedanib.

Pirfenidone			Nintedanib		
**Total** (552)	**N**	**P(%)**	**Total** (656)	**N**	**P(%)**
**Age** (295)			**Age** (467)		
<65	94	31.864%	<65	161	34.475%
≥65, <75	114	38.644%	≥65, <75	152	32.549%
≥75	87	29.492%	≥75	154	32.976%
**Gender** (546)			**Gender** (606)		
Male	375	68.681%	Male	397	65.512%
Female	171	31.319%	Female	209	34.488%
**Outcome** (544)			**Outcome** (652)		
Other	213	39.154%	Hospitalization	272	41.718%
Hospitalization	162	29.779%	Death	177	27.147%
Death	142	26.103%	Other	177	27.147%
Life-Threatening	17	3.125%	Life-Threatening	19	2.9145%
Disability	10	1.838%	Disability	7	1.704%
**PT**	N	P(%)	**PT**	N	P(%)
Pulmonary fibrosis	143	24.826%	Pulmonary hypertension	138	19.382%
Pulmonary hypertension	98	17.014%	Pulmonary fibrosis	94	13.202%
Deafness	57	9.896%	Febrile neutropenia	62	8.708%
Pulmonary arterial hypertension	41	7.118%	Acute kidney injury	54	7.584%
Renal failure	37	6.424%	Pancreatitis	49	6.882%
Pancreatitis	31	5.382%	Renal failure	47	6.601%
Blindness	22	3.819%	Drug-induced liver injury	46	6.461%
Acute kidney injury	22	3.819%	Pulmonary arterial hypertension	29	4.073%
Hepatic failure	16	2.778%	Intestinal perforation	28	3.933%
Angioedema	14	2.431%	Hepatic failure	27	3.792%
Dermatitis exfoliative generalised	11	1.910%	Neutropenic sepsis	26	3.652%
Acute hepatic failure	10	1.736%	Deafness	16	2.247%
Drug reaction with eosinophilia and systemic symptoms	9	1.563%	Pancreatitis acute	15	2.107%
Drug-induced liver injury	9	1.563%	Rhabdomyolysis	12	1.685%
Stevens-Johnson syndrome	6	1.042%	Blindness	11	1.545%
Pancytopenia	6	1.042%	Pancytopenia	8	1.124%
Pancreatitis acute	6	1.042%	Haemolytic anaemia	7	0.983%
Erythema multiforme	6	1.042%	Acute hepatic failure	5	0.702%
Anaphylactic reaction	5	0.868%	Dermatitis exfoliative generalised	5	0.702%
Intestinal perforation	4	0.694%	Angioedema	5	0.702%
Rhabdomyolysis	4	0.694%	Agranulocytosis	4	0.562%
Toxic epidermal necrolysis	3	0.521%	Aplastic anaemia	3	0.421%
Agranulocytosis	3	0.521%	Ventricular fibrillation	2	0.281%
Sudden cardiac death	2	0.347%	Anaphylactic reaction	2	0.281%
Bone marrow failure	2	0.347%	Deafness transitory	2	0.281%
Anaphylactic shock	2	0.347%	Optic ischaemic neuropathy	2	0.281%
Febrile neutropenia	2	0.347%	Stevens-Johnson syndrome	2	0.281%
Haemolytic anaemia	1	0.174%	Sudden cardiac death	2	0.281%
Sudden hearing loss	1	0.174%	Aplasia pure red cell	1	0.140%
Deafness neurosensory	1	0.174%	Autoimmune haemolytic anaemia	1	0.140%
Azotaemia	1	0.174%	Drug reaction with eosinophilia and systemic symptoms	1	0.140%
Anaphylactoid reaction	1	0.174%	Erythema multiforme	1	0.140%
Total	576^*^		Granulocytopenia	1	0.140%
			Haemolysis	1	0.140%
			Hepatic necrosis	1	0.140%
			Immune thrombocytopenia	1	0.140%
			Sudden hearing loss	1	0.140%
			Total	712^*^	

^*^More than one adverse reaction may occur in the same patient.

## Discussion

This study compared the AE reporting trends and characteristics of PFD and NDN. The number of reported AEs related to these two drugs increased significantly in 2015.

In that year, PFD and NDN were listed as novel agents conditionally recommended for the treatment of IPF by ATS/ERS/JRS/ALAT (Raghu et al., 2015). However, the total and annual numbers of AEs were higher for PFD than for NDN. Although both drugs were approved for the treatment of IPF by the FDA in 2014, PFD was approved for the treatment of mild-to-moderate IPF in adults in the European Union in 2011. At the same time, some studies found that PFD had significantly lower weighted annual mean anti-fibrotic drug costs than NDN (Corral et al., 2020). Based on this situation, PFD might be more widely used in more patients than NDN, thereby leading to a larger number of reported AEs. In terms of reporting regions, the number of reported AEs was significantly higher for the US than for other countries. This could be related to the higher incidence of IPF in North America and Europe than in Asia and South America (Hutchinson et al., 2015). Events were more common in males than in patients aged ≥65 years. This is explainable by the fact that male sex and older age have been identified as risk factors for IPF (Jee, et al., 2021).

Disproportionality analysis revealed that gastrointestinal disorders were the most commonly SOC for both drugs, which corresponded to previous findings. Regarding gastrointestinal disorders, the most frequently PTs were nausea (N = 3354, ROR = 4.437, 95% CI = 4.278–4.602) and diarrhea (N = 2933, ROR = 16.816, 95% CI = 16.102–17.562) for PFD and NDN, respectively. The clinical safety profile of PFD was demonstrated in the CAPACITY studies, which detected nausea in 36% of PFD-treated patients. However, the rate of treatment discontinuation related to nausea was only 1.4% (Noble et al., 2011). To prevent gastrointestinal AEs, it is recommended to take PFD during or after a meal (European Medicine Agency, 2023). A longer initial dosing titration scheme has been found to further improve the tolerability of PFD (Clinical trials.gov, 2016, NCT01933334). In the INPULSIS trials, diarrhea was the most commonly reported AE, being experienced by 62% of NDN-treated patients (Richeldi et al., 2014). However, only 4.4% of patients on NDN permanently discontinued treatment because of diarrhea (European Medicines Agency, 2015). Loperamide is currently recommended for the management of NDN-associated diarrhea (Corte et al., 2015).

“Eye disorders” represented the most statistically significant SOC for PFD, but the total number of reports was limited. “Cardiac disorders” represented the most statistically significant SOC for NDN, and it was mainly represented by atrial fibrillation (N = 82, ROR = 2.129, 95% CI = 1.713–2.647), cardiac failure (N = 72, ROR = 2.136, 95% CI = 1.694–2.694), and pericardial effusion (N = 24, ROR = 2.654, 95% CI = 1.777–3.964). The phase II TOMORROW trial and the two phase III INPULSIS trials evaluated the most frequent AEs, defined as those with an incidence of more than 10% in the NDN and placebo groups. Cardiac disorders were not the most frequent AEs in all the aforementioned three trials (Richeldi et al., 2011; Richeldi et al., 2014). A subgroup analysis of data from the open-label extension study (INPULSIS^®^-ON) of the INPULSIS^®^ trials focused on the long-term safety of NDN in Asian patients. In this trial, major adverse cardiovascular events were observed at event rates of 5.5 and 2.8 events per 100 exposure-years among patients who continued and initiated NDN, respectively (Song et al., 2020). From the safety-related labeling changes approved by the FDA Center for Drug Evaluation and Research, arterial thromboembolic events, especially myocardial infarction, were mentioned in the “warnings and precautions” section (https://www.accessdata.fda.gov/scripts/cder/safetylabelingchanges/index.cfm?event=searchdetail.page&DrugNameID=839#). A multi-center phase II study including 62 Chinese patients with advanced non-small-cell lung cancer who received NDN as second-line therapy recorded a heart failure rate of 69.35% (43/62) (Dai et al., 2015). Therefore, cardiac disorders should be closely monitored in clinical practice.

“Elevated liver enzymes and drug-induced liver injury” were listed in the “warnings and precautions” sections for both PFD and NDN. The prescribing information recommends that liver function should be monitored prior to initiating NDN and periodically during treatment. In 2020, the UK government published a drug safety update concerning risk of serious liver injury related to NDN, including two fatal outcomes (Verma et al., 2017; Benesic et al., 2019). The LiverTox^®^ database, which was produced by the National Institute of Diabetes and Digestive and Kidney Diseases, uses a five-point scale to estimate whether a medication is a cause of liver injury (Evans et al., 2001; LiverTox, 2012; Hutchinson et al., 2015; Corral et al., 2020; Neha et al., 2020; Jee, et al., 2021). In that database, NDN was assigned a likelihood score of E^*^ (unproven but suspected cause of clinically apparent liver injury), and PFD was assigned a likelihood score of D (possible rare cause of clinically apparent liver injury) (Nintedanib, 2017; LiverTox, 2020). In our study, liver injury associated with PFD was represented by hepatic failure (16, 2.778%), acute hepatic failure (10, 1.736%), and drug-induced liver injury (9, 1.563%). Simultaneously, NDN was associated with drug-induced liver injury (46, 6.461%), hepatic failure (27, 3.792%), acute hepatic failure (5, 0.702%), and hepatic necrosis (1, 0.140%). Therefore, NDN-associated liver injury should be further evaluated, and the database should be updated according to the evaluation results.

The disproportional analysis method used in this study has many advantages in pharmacovigilance studies, although it also has certain limitations. First of all, ADR signals detected by disproportional analysis indicate that there is a statistical association between adverse drug reaction events, which has a certain suggestive effect, however, the determination of causality needs further clinical studies to verify. Second, this approach is unable to include all factors in the analysis, due to the spontaneity of the reporting system and the absence of partial information. Third, although the OpenVigil 2.1 software provides an easy way to access, extract, and analyze the open FDA interface, it does not take dosage into account. Finally, reports of adverse reactions to both drugs came mainly from the United States, with limited reports from Asian populations. Further research is needed to determine whether there are differences in adverse effects between different ethnic groups.

## Conclusion

The results of our study highlighted the adverse reactions and potential safety issues of PFD and NDN. The findings included associations of PFD with eye disorders and NDN with cardiovascular disorders, which were rarely mentioned or not reported in the medicine specification and previous reports. In addition, liver injury was more frequently associated with NDN than with PFD. Clinicians and regulators should pay more attention to the signals of related adverse reactions that occur frequently. These findings should be validated to guide clinicians, regulators, and the industry to focus on the most relevant signals beyond the information already contained in product characteristic summaries. Our research results could help clinicians identify the risks related to clinical drug use in the future and guide the rational and safe use of NDN and PFD. Further detailed analyses are required characterize the nature of the identified signals and investigate other correlations.

## Data Availability

The original contributions presented in the study are included in the article/Supplementary Material, further inquiries can be directed to the corresponding authors.
