# Supplementary material for: Comparison of the safety profiles for pirfenidone and nintedanib: a disproportionality analysis of the US food and drug administration adverse event reporting system
Source: Front Pharmacol. 2024 May 27;15:1256649. doi: 10.3389/fphar.2024.1256649 (PMC11163030; doi:10.3389/fphar.2024.1256649)
Supplement: Supplementary file 1 [file Table1.docx]

**Table S1 Number of PT signals occurring with pirfenidone and nintedanib**

| Algorithms | Pirfenidone | Nintedanib |
| --- | --- | --- |
| ROR (PT/N) | 287 | 596 |
| PRR (PT/N) | 197 | 425 |

**Table S2 Disproportionality analysis of pirfenidone and nintedanib.**

|  | Pirfenidone | | | | | Nintedanib | | | | |
| --- | --- | --- | --- | --- | --- | --- | --- | --- | --- | --- |
| SOC | PT | N | χ2 | PRR | ROR (95CI) | PT (N) | N | χ2 | PRR | ROR (95CI) |
| Blood and lymphatic system | increased tendency to bruise | 20 | 11.603 | 2.183 | 2.184 (1.407-3.390) | febrile neutropenia | 46 | 28.262 | 2.181 | 2.187 (1.636-2.932) |
|  |  |  |  |  |  | increased tendency to bruise | 21 | 79.504 | 5.864 | 5.874(3.824-9.025) |
|  |  |  |  |  |  | haemorrhagic diathesis | 18 | 174.797 | 12.367 | 12.388 (7.78-19.725) |
|  |  |  |  |  |  | splenic vein thrombosis | 8 | 274.067 | 42.697 | 42.731 (21.063-86.688) |
|  |  |  |  |  |  | white blood cell disorder | 4 | 4.969 | 3.546 | 3.547(1.329-9.468) |
|  |  |  |  |  |  | haemorrhagic disorder | 3 | 9.132 | 6.595 | 6.597(2.12-20.531) |
| Cardiac disorders | heart valve incompetence | 9 | 10.328 | 3.046 | 3.047 (1.581-5.871) | atrial fibrillation | 82 | 47.484 | 2.12 | 2.129 (1.713-2.647) |
|  | aortic valve calcification | 4 | 20.522 | 8.996 | 8.997 (3.34-24.235) | cardiac failure | 72 | 41.969 | 2.128 | 2.136 (1.694-2.694) |
|  | mitral valve calcification | 3 | 5.444 | 4.744 | 4.744 (1.52-14.809) | pericardial effusion | 24 | 22.996 | 2.65 | 2.654 (1.777-3.964) |
|  |  |  |  |  |  | pulseless electrical activity | 13 | 69.984 | 7.808 | 7.817 (4.528-13.494) |
|  |  |  |  |  |  | right ventricular failure | 12 | 23.062 | 3.908 | 3.912 (2.218-6.898) |
|  |  |  |  |  |  | acute coronary syndrome | 12 | 14.646 | 3.064 | 3.067 (1.739-5.406) |
|  |  |  |  |  |  | atrioventricular block | 11 | 13.258 | 3.063 | 3.066 (1.696-5.542) |
|  |  |  |  |  |  | arteriosclerosis coronary artery | 7 | 4.268 | 2.374 | 2.375 (1.131-4.987) |
|  |  |  |  |  |  | heart valve incompetence | 6 | 16.198 | 5.181 | 5.184 (2.324-11.565) |
|  |  |  |  |  |  | aortic valve calcification | 4 | 62.238 | 23.009 | 23.018 (8.544-62.01) |
|  |  |  |  |  |  | cardiac ventricular thrombosis | 4 | 30.809 | 12.363 | 12.368 (4.614-33.152) |
|  |  |  |  |  |  | aortic valve stenosis | 4 | 10.555 | 5.515 | 5.517 (2.065-14.74) |
|  |  |  |  |  |  | paroxysmal arrhythmia | 3 | 170.269 | 91.359 | 91.387 (28.603-297.597) |
|  |  |  |  |  |  | mitral valve calcification | 3 | 20.28 | 12.134 | 12.137 (3.888-37.889) |
|  |  |  |  |  |  | cor pulmonale | 3 | 8.013 | 6.031 | 6.033 (1.939-18.711) |
|  |  |  |  |  |  | cardiac fibrillation | 3 | 4.885 | 4.431 | 4.432 (1.426-13.778) |
| Gastrointestinal disorders | nausea | 3354 | 7659.632 | 3.978 | 4.437 (4.278-4.602) | diarrhoea | 2933 | 30244.102 | 12.093 | 16.816 (16.102-17.562) |
|  | diarrhoea | 1999 | 3093.293 | 3.21 | 3.402 (3.249-3.561) | nausea | 1337 | 3139.429 | 4.032 | 4.509 (4.256-4.778) |
|  | vomiting | 1088 | 732.335 | 2.213 | 2.268 (2.134-2.41) | vomiting | 769 | 1750.491 | 3.994 | 4.248 (3.946-4.573) |
|  | abdominal discomfort | 1078 | 4806.163 | 6.345 | 6.585 (6.192-7.002) | constipation | 518 | 2458.207 | 6.597 | 6.908 (6.322-7.549) |
|  | abdominal pain upper | 939 | 2491.458 | 4.438 | 4.572 (4.282-4.881) | abdominal pain upper | 508 | 2171.782 | 6.11 | 6.389 (5.842-6.988) |
|  | dyspepsia | 745 | 3961.305 | 7.261 | 7.452 (6.924-8.021) | abdominal discomfort | 449 | 2171.616 | 6.698 | 6.971 (6.339-7.665) |
|  | constipation | 461 | 335.885 | 2.294 | 2.318 (2.113-2.542) | abdominal pain | 277 | 399.599 | 3.121 | 3.183 (2.824-3.587) |
|  | gastrooesophageal reflux disease | 410 | 1285.324 | 4.971 | 5.037 (4.566-5.557) | flatulence | 255 | 2372.306 | 11.336 | 11.611 (10.246-13.157) |
|  | gastric disorder | 375 | 2039.125 | 7.408 | 7.505 (6.771-8.319) | gastric disorder | 173 | 1156.526 | 8.656 | 8.793 (7.561-10.226) |
|  | gastrointestinal disorder | 215 | 205.93 | 2.581 | 2.594 (2.267-2.968) | gastrointestinal disorder | 162 | 508.344 | 4.966 | 5.032 (4.307-5.879) |
|  | abdominal distension | 211 | 117.467 | 2.082 | 2.091 (1.826-2.396) | haematochezia | 150 | 965.695 | 8.409 | 8.524 (7.25-10.022) |
|  | flatulence | 194 | 317.822 | 3.363 | 3.381 (2.934-3.897) | dyspepsia | 142 | 249.865 | 3.489 | 3.526 (2.986-4.162) |
|  | eructation | 69 | 146.984 | 3.938 | 3.946 (3.112-5.004) | abdominal distension | 131 | 207.887 | 3.3 | 3.331 (2.803-3.959) |
|  | hiatus hernia | 42 | 63.642 | 3.275 | 3.279 (2.42-4.444) | gastrooesophageal reflux disease | 117 | 216.761 | 3.597 | 3.628 (3.023-4.355) |
|  | frequent bowel movements | 39 | 21.927 | 2.122 | 2.124 (1.55-2.91) | rectal haemorrhage | 113 | 526.297 | 6.576 | 6.641 (5.514-7.999) |
|  | oesophageal pain | 28 | 114.711 | 6.18 | 6.186 (4.258-8.987) | gastrointestinal haemorrhage | 110 | 97.797 | 2.501 | 2.518 (2.086-3.039) |
|  | faeces soft | 23 | 76.011 | 5.348 | 5.352 (3.546-8.077) | dry mouth | 89 | 93.91 | 2.704 | 2.719 (2.206-3.351) |
|  | hyperchlorhydria | 23 | 10.813 | 2.021 | 2.022 (1.342-3.046） | faeces discoloured | 75 | 484.563 | 8.49 | 8.547 (6.804-10.737) |
|  | anorectal discomfort | 19 | 34.874 | 3.725 | 3.727 (2.372-5.856) | faeces soft | 68 | 2546.536 | 41.514 | 41.796 (32.773-53.305) |
|  | epigastric discomfort | 16 | 33.332 | 4.043 | 4.045 (2.472-6.62) | frequent bowel movements | 48 | 225.259 | 6.688 | 6.716 (5.053-8.926) |
|  | gastrointestinal motility disorder | 15 | 20.664 | 3.223 | 3.224 (1.94-5.36) | diarrhoea haemorrhagic | 47 | 546.673 | 13.988 | 14.05 (10.529-18.749) |
|  | chapped lips | 14 | 16.544 | 2.995 | 2.997 (1.771-5.07) | colitis | 44 | 77.246 | 3.534 | 3.545 (2.635-4.769) |
|  | bowel movement irregularity | 13 | 5.721 | 2.024 | 2.025 (1.174-3.492） | haemorrhoids | 42 | 157.536 | 5.705 | 5.725 (4.225-7.758) |
|  | intestinal polyp | 8 | 22.57 | 5.165 | 5.166 (2.572-10.378) | retching | 38 | 99.542 | 4.498 | 4.511 (3.278-6.208) |
|  | trichoglossia | 6 | 63.517 | 15.18 | 15.184 (6.72-34.307) | gastric haemorrhage | 36 | 132.643 | 5.654 | 5.671 (4.084-7.873) |
|  | faeces pale | 6 | 4.387 | 2.6 | 2.601 (1.165-5.805) | eructation | 32 | 87.644 | 4.647 | 4.659 (3.29-6.597) |
|  | defaecation disorder | 4 | 22.888 | 9.814 | 9.815 (3.64-26.464) | haematemesis | 32 | 39.426 | 2.956 | 2.963 (2.093-4.194) |
|  | oral discharge | 3 | 11.878 | 8.043 | 8.043 (2.565-25.222) | intestinal haemorrhage | 29 | 284.096 | 12.211 | 12.244 (8.486-17.667) |
|  | gastric varices haemorrhage | 3 | 11.178 | 7.686 | 7.687 (2.453-24.093) | intestinal perforation | 27 | 102.828 | 5.834 | 5.847 (4.004-8.54) |
|  | gastric varices | 3 | 9.558 | 6.861 | 6.862 (2.192-21.482) | gastrointestinal sounds abnormal | 26 | 345.147 | 15.986 | 16.026 (10.874-23.619) |
|  | oesophageal achalasia | 3 | 5.374 | 4.707 | 4.708 (1.508-14.693) | intestinal obstruction | 26 | 12.532 | 2.028 | 2.031 (1.381-2.985) |
|  |  |  |  |  |  | melaena | 25 | 23.496 | 2.624 | 2.628 (1.774-3.894) |
|  |  |  |  |  |  | abdominal pain lower | 22 | 10.733 | 2.046 | 2.048 (1.348-3.114) |
|  |  |  |  |  |  | haemorrhoidal haemorrhage | 21 | 186.983 | 11.402 | 11.424 (7.428-17.571) |
|  |  |  |  |  |  | intestinal ischaemia | 20 | 124.692 | 8.539 | 8.555 (5.507-13.29) |
|  |  |  |  |  |  | bowel movement irregularity | 18 | 89.099 | 7.182 | 7.193 (4.523-11.44) |
|  |  |  |  |  |  | anal incontinence | 17 | 76.917 | 6.736 | 6.746 (4.186-10.873) |
|  |  |  |  |  |  | gastric ulcer | 17 | 8.815 | 2.108 | 2.11 (1.311-3.397) |
|  |  |  |  |  |  | hiatus hernia | 17 | 26.007 | 3.375 | 3.379 (2.098-5.441) |
|  |  |  |  |  |  | colitis ischaemic | 16 | 56.6 | 5.666 | 5.673 (3.47-9.277) |
|  |  |  |  |  |  | gastrointestinal pain | 15 | 28.166 | 3.804 | 3.808 (2.293-6.325) |
|  |  |  |  |  |  | gastrointestinal motility disorder | 12 | 51.068 | 6.584 | 6.591 (3.735-11.631) |
|  |  |  |  |  |  | large intestine perforation | 12 | 27.925 | 4.383 | 4.387 (2.487-7.736) |
|  |  |  |  |  |  | gastrointestinal perforation | 11 | 38.834 | 5.802 | 5.807 (3.21-10.507) |
|  |  |  |  |  |  | abnormal faeces | 11 | 20.863 | 3.903 | 3.906 (2.16-7.064) |
|  |  |  |  |  |  | lower gastrointestinal haemorrhage | 11 | 15.212 | 3.283 | 3.286 (1.817-5.941) |
|  |  |  |  |  |  | mesenteric vein thrombosis | 10 | 192.288 | 23.857 | 23.881 (12.754-44.714) |
|  |  |  |  |  |  | large intestinal haemorrhage | 10 | 84.754 | 11.504 | 11.515 (6.173-21.482) |
|  |  |  |  |  |  | gastric perforation | 10 | 57.097 | 8.35 | 8.358 (4.484-15.577) |
|  |  |  |  |  |  | large intestine polyp | 10 | 24.615 | 4.604 | 4.608 (2.475-8.578) |
|  |  |  |  |  |  | gastric ulcer haemorrhage | 10 | 20.341 | 4.097 | 4.101 (2.203-7.633) |
|  |  |  |  |  |  | oesophagitis | 10 | 6.195 | 2.314 | 2.316 (1.245-4.308) |
|  |  |  |  |  |  | enterocolitis | 9 | 22.791 | 4.733 | 4.736 (2.46-9.119) |
|  |  |  |  |  |  | duodenal ulcer | 8 | 7.061 | 2.722 | 2.723 (1.36-5.452) |
|  |  |  |  |  |  | diverticulum | 8 | 5.646 | 2.479 | 2.48 (1.239-4.966) |
|  |  |  |  |  |  | regurgitation | 7 | 18.362 | 4.978 | 4.981 (2.37-10.469) |
|  |  |  |  |  |  | food poisoning | 7 | 13.281 | 4.079 | 4.081 (1.942-8.575) |
|  |  |  |  |  |  | faeces pale | 6 | 23.284 | 6.651 | 6.655 (2.981-14.855) |
|  |  |  |  |  |  | pneumatosis intestinalis | 6 | 15.892 | 5.117 | 5.12 (2.295-11.422) |
|  |  |  |  |  |  | functional gastrointestinal disorder | 6 | 12.609 | 4.425 | 4.427 (1.985-9.873) |
|  |  |  |  |  |  | epigastric discomfort | 6 | 9.949 | 3.854 | 3.856 (1.729-8.597) |
|  |  |  |  |  |  | defaecation urgency | 6 | 8.025 | 3.432 | 3.434 (1.54-7.655) |
|  |  |  |  |  |  | coating in mouth | 5 | 98.912 | 27.247 | 27.261 (11.215-66.265) |
|  |  |  |  |  |  | discoloured vomit | 5 | 60.165 | 17.257 | 17.265 (7.132-41.792) |
|  |  |  |  |  |  | obstruction gastric | 5 | 32.518 | 10.211 | 10.216 (4.233-24.656) |
|  |  |  |  |  |  | diverticulum intestinal haemorrhagic | 5 | 12.787 | 5.156 | 5.159 (2.142-12.423) |
|  |  |  |  |  |  | anal haemorrhage | 5 | 12.308 | 5.031 | 5.033 (2.09-12.121) |
|  |  |  |  |  |  | duodenal ulcer haemorrhage | 5 | 8.271 | 3.961 | 3.963 (1.646-9.538) |
|  |  |  |  |  |  | proctitis | 4 | 8.178 | 4.69 | 4.692 (1.757-12.5320 |
|  |  |  |  |  |  | diverticular perforation | 4 | 7.462 | 4.439 | 4.44 (1.663-11.859) |
|  |  |  |  |  |  | abdominal rigidity | 4 | 6.132 | 3.967 | 3.968 (1.486-10.595) |
|  |  |  |  |  |  | vomiting projectile | 4 | 5.322 | 3.675 | 3.676 (1.377-9.814) |
|  |  |  |  |  |  | faeces hard | 4 | 4.611 | 3.414 | 3.415 (1.279-9.117) |
|  |  |  |  |  |  | oesophageal stenosis | 4 | 4.566 | 3.398 | 3.399 (1.273-9.072) |
|  |  |  |  |  |  | jejunal perforation | 3 | 63.338 | 33.763 | 33.773 (10.693-106.671) |
|  |  |  |  |  |  | saliva altered | 3 | 13.911 | 8.977 | 8.98 (2.882-27.985) |
|  |  |  |  |  |  | oesophageal rupture | 3 | 11.671 | 7.864 | 7.866 (2.526-24.498) |
|  |  |  |  |  |  | intestinal mass | 3 | 8.967 | 6.512 | 6.514 (2.093-20.272) |
|  |  |  |  |  |  | tongue coated | 3 | 5.835 | 4.923 | 4.924 (1.583-15.311) |
|  |  |  |  |  |  | large intestinal obstruction | 3 | 5.008 | 4.495 | 4.496 (1.446-13.978) |
| General disorders and administration site conditions | death | 4626 | 10654.426 | 3.965 | 4.636 (4.49-4.787) | death | 1097 | 924.191 | 2.387 | 2.562 (2.406-2.728) |
|  | fatigue | 2356 | 3279.264 | 3.029 | 3.24 (3.105-3.381) | fatigue | 784 | 769.571 | 2.566 | 2.701 (2.511-2.906) |
|  | no adverse event | 816 | 2036.548 | 4.273 | 4.383 (4.086-4.701) | asthenia | 490 | 819.205 | 3.359 | 3.483 (3.18-3.815) |
|  | asthenia | 792 | 476.56 | 2.127 | 2.164 (2.016-2.323) | chest pain | 206 | 191.41 | 2.537 | 2.57 (2.238-2.951) |
|  | drug intolerance | 237 | 185.764 | 2.369 | 2.382 (2.095-2.708) | disease progression | 135 | 139.683 | 2.67 | 2.693 (2.272-3.192) |
|  | ill-defined disorder | 146 | 160.395 | 2.752 | 2.763 (2.347-3.253) | general physical health deterioration | 123 | 180.52 | 3.168 | 3.196 (2.674-3.819) |
|  | illness | 108 | 53.771 | 2.011 | 2.016 (1.668-2.436) | illness | 108 | 355.5 | 5.145 | 5.191 (4.292-6.278) |
|  | fibrosis | 28 | 140.769 | 7.197 | 7.204 (4.956-10.469) | drug intolerance | 103 | 102.106 | 2.625 | 2.642 (2.175-3.209) |
|  | secretion discharge | 25 | 25.002 | 2.704 | 2.705 (1.825-4.01) | peripheral swelling | 103 | 54.51 | 2.05 | 2.061 (1.697-2.503) |
|  | exercise tolerance decreased | 23 | 50.713 | 4.118 | 4.121 (2.732-6.215) | chest discomfort | 90 | 66.491 | 2.321 | 2.334 (1.896-2.873) |
|  | crepitations | 14 | 32.406 | 4.343 | 4.345 (2.566-7.358) | secretion discharge | 39 | 334.781 | 10.829 | 10.868 (7.923-14.909) |
|  | systemic inflammatory response syndrome | 13 | 20.515 | 3.492 | 3.493 (2.023-6.031) | feeling cold | 26 | 18.626 | 2.341 | 2.344 (1.595-3.446) |
|  |  |  |  |  |  | mucosal inflammation | 21 | 12.989 | 2.228 | 2.23 (1.453-3.424) |
|  |  |  |  |  |  | hernia | 19 | 18.388 | 2.684 | 2.687 (1.712-4.217) |
|  |  |  |  |  |  | ulcer haemorrhage | 18 | 70.403 | 6.043 | 6.052 (3.807-9.624) |
|  |  |  |  |  |  | sudden death | 13 | 9.492 | 2.425 | 2.427 (1.408-4.184) |
|  |  |  |  |  |  | decreased activity | 12 | 12.365 | 2.826 | 2.829 (1.605-4.986) |
|  |  |  |  |  |  | performance status decreased | 11 | 35.379 | 5.442 | 5.447 (3.011-9.854) |
|  |  |  |  |  |  | fibrosis | 10 | 40.943 | 6.5 | 6.505 (3.492-12.118) |
|  |  |  |  |  |  | exercise tolerance decreased | 10 | 24.185 | 4.553 | 4.557 (2.448-8.483) |
|  |  |  |  |  |  | terminal state | 8 | 4.638 | 2.3 | 2.301 (1.149-4.605) |
|  |  |  |  |  |  | disease complication | 7 | 10.927 | 3.653 | 3.655 (1.74-7.679) |
|  |  |  |  |  |  | drug tolerance decreased | 4 | 10.577 | 5.522 | 5.524 (2.067-14.76) |
|  |  |  |  |  |  | mucosal discolouration | 3 | 20.184 | 12.086 | 12.09 (3.873-37.74) |
|  |  |  |  |  |  | perforated ulcer | 3 | 12.251 | 8.153 | 8.155 (2.618-25.403) |
|  |  |  |  |  |  | catheter site erythema | 3 | 7.653 | 5.85 | 5.851 (1.881-18.204) |
| Hepatobiliary disorders |  |  |  |  |  | liver disorder | 107 | 403.3 | 5.649 | 5.7 (4.709-6.900) |
|  |  |  |  |  |  | hepatic function abnormal | 51 | 90.68 | 3.549 | 3.563 (2.704-4.694) |
|  |  |  |  |  |  | hepatotoxicity | 51 | 163.033 | 5.093 | 5.114 (3.881-6.738) |
|  |  |  |  |  |  | liver injury | 44 | 144.295 | 5.195 | 5.214 (3.875-7.017) |
|  |  |  |  |  |  | drug-induced liver injury | 32 | 37.921 | 2.901 | 2.907 (2.054-4.115) |
|  |  |  |  |  |  | hepatic cirrhosis | 26 | 44.038 | 3.508 | 3.515 (2.391-5.169) |
|  |  |  |  |  |  | hepatitis | 25 | 16.758 | 2.284 | 2.287 (1.544-3.388) |
|  |  |  |  |  |  | hepatic steatosis | 17 | 12.765 | 2.42 | 2.423 (1.505-3.901) |
|  |  |  |  |  |  | cholecystitis | 11 | 5.922 | 2.185 | 2.186 (1.21-3.951) |
|  |  |  |  |  |  | portal vein thrombosis | 9 | 35.43 | 6.383 | 6.388 (3.316-12.305) |
|  |  |  |  |  |  | cholangitis | 6 | 5.347 | 2.824 | 2.825 (1.267-6.296) |
|  |  |  |  |  |  | hepatitis toxic | 5 | 9.907 | 4.398 | 4.400 (1.828-10.594) |
|  |  |  |  |  |  | cholestatic liver injury | 4 | 10.86 | 5.62 | 5.621 (2.104-15.021) |
|  |  |  |  |  |  | biliary obstruction | 3 | 22.022 | 12.997 | 13.000 (4.163-40.603) |
| Infections and infestations | pneumonia | 919 | 1316.078 | 3.106 | 3.186 (2.983-3.404) | pneumonia | 403 | 711.864 | 3.469 | 3.575 (3.235-3.95) |
|  | lower respiratory tract infection | 128 | 207.893 | 3.356 | 3.368 (2.829-4.009) | nasopharyngitis | 157 | 127.347 | 2.4 | 2.423 (2.069-2.837) |
|  | lung infection | 59 | 74.902 | 2.975 | 2.980 (2.306-3.851) | covid-19 | 141 | 249.142 | 3.497 | 3.534 (2.992-4.174) |
|  | respiratory tract infection | 50 | 32.716 | 2.234 | 2.237 (1.694-2.954) | bronchitis | 71 | 67.577 | 2.587 | 2.599 (2.057-3.283) |
|  | oral candidiasis | 23 | 11.854 | 2.084 | 2.085 (1.384-3.142) | diverticulitis | 66 | 302.655 | 6.541 | 6.578 95.16-8.386） |
|  | covid-19 pneumonia | 18 | 10.636 | 2.206 | 2.207 (1.388-3.508) | respiratory tract infection | 36 | 81.329 | 4.108 | 4.12 (2.968-5.718) |
|  | gastric infection | 17 | 53.186 | 5.221 | 5.224 (3.237-8.431) | lung infection | 34 | 84.776 | 4.372 | 4.383 (3.128-6.142) |
|  | coronavirus infection | 14 | 9.541 | 2.355 | 2.355 (1.393-3.983) | pneumonia bacterial | 34 | 348.748 | 12.64 | 12.681 (9.037-17.794) |
|  | viral upper respiratory tract infection | 10 | 4.068 | 2.006 | 2.006 (1.078-3.735) | lower respiratory tract infection | 32 | 18.117 | 2.132 | 2.136 (1.509-3.023) |
|  | rhinocerebral mucormycosis | 4 | 46.798 | 18.194 | 18.196 (6.682-49.555) | cystitis | 28 | 18.235 | 2.252 | 2.255 (1.556-3.269) |
|  |  |  |  |  |  | neutropenic sepsis | 19 | 80.3 | 6.373 | 6.383 (4.064-10.026) |
|  |  |  |  |  |  | clostridium difficile infection | 17 | 21.3 | 3.045 | 3.049 (1.893-4.91) |
|  |  |  |  |  |  | covid-19 pneumonia | 15 | 39.87 | 4.698 | 4.703 (2.831-7.814) |
|  |  |  |  |  |  | coronavirus infection | 13 | 44.278 | 5.59 | 5.596 (3.243-9.655) |
|  |  |  |  |  |  | gastrointestinal infection | 12 | 26.342 | 4.229 | 4.233 (2.4-7.465) |
|  |  |  |  |  |  | oral candidiasis | 12 | 11.876 | 2.775 | 2.777 (1.575-4.895) |
|  |  |  |  |  |  | rhinitis | 8 | 8.644 | 2.985 | 2.987 (1.492-5.98) |
|  |  |  |  |  |  | dysentery | 7 | 43.444 | 9.292 | 9.298 (4.417-19.574) |
|  |  |  |  |  |  | helicobacter infection | 7 | 10.522 | 3.579 | 3.581 (1.705-7.523) |
|  |  |  |  |  |  | post procedural infection | 7 | 6.675 | 2.857 | 2.858 (1.361-6.003) |
|  |  |  |  |  |  | diverticulitis intestinal haemorrhagic | 4 | 59.714 | 22.148 | 22.156 (8.228-59.665) |
|  |  |  |  |  |  | haemophilus infection | 4 | 29.952 | 12.075 | 12.079 (4.507-32.375) |
|  |  |  |  |  |  | pneumonia viral | 4 | 7.288 | 4.378 | 4.379 (1.64-11.695) |
|  |  |  |  |  |  | post procedural pneumonia | 4 | 104.58 | 37.651 | 37.666 (13.886-102.167) |
|  |  |  |  |  |  | pulmonary sepsis | 4 | 21.364 | 9.183 | 9.187 (3.432-24.588) |
|  |  |  |  |  |  | gastrointestinal bacterial infection | 3 | 17.482 | 10.748 | 10.751 (3.447-33.537) |
|  |  |  |  |  |  | pneumococcal infection | 3 | 22.697 | 13.331 | 13.335 (4.269-41.656) |
|  |  |  |  |  |  | pneumonia pseudomonal | 3 | 13.112 | 8.581 | 8.583 (2.755-26.742) |
|  |  |  |  |  |  | pneumonia respiratory syncytial viral | 3 | 24.03 | 13.992 | 13.996 (4.479-43.736) |
|  |  |  |  |  |  | pneumonia staphylococcal | 3 | 4.483 | 4.22 | 4.221 (1.358-13.121) |
| Injury, poisoning, and procedural complications | intentional product use issue | 437 | 2015.802 | 6.534 | 6.632 (6.029-7.296) | contusion | 90 | 73.821 | 2.422 | 2.435 (1.978-2.997) |
|  | sunburn | 233 | 7157.541 | 35.526 | 35.849 (31.338-41.01) | sunburn | 13 | 33.902 | 4.682 | 4.687 (2.717-8.084) |
|  | product dose omission in error | 95 | 711.538 | 9.653 | 9.686 (7.900-11.876) | intentional dose omission | 11 | 5.296 | 2.103 | 2.104 (1.164-3.802) |
|  | muscle strain | 33 | 67.699 | 3.901 | 3.905 (2.771-5.504) | post procedural haemorrhage | 11 | 4.753 | 2.029 | 2.031 (1.124-3.670) |
|  | thermal burn | 24 | 28.267 | 2.923 | 2.925 (1.957-4.371) | muscle strain | 10 | 11.407 | 3.004 | 3.006 (1.615-5.593) |
|  | back injury | 23 | 15.501 | 2.298 | 2.299 (1.526-3.464) | skin laceration | 10 | 8.712 | 2.656 | 2.657 (1.428-4.944) |
|  | chest injury | 19 | 152.898 | 10.668 | 10.675 (6.768-16.838) | back injury | 9 | 5.315 | 2.292 | 2.293 (1.192-4.412) |
|  | intercepted product storage error | 8 | 5.603 | 2.476 | 2.476 (1.236-4.963) | wrong dose | 9 | 61.434 | 9.727 | 9.735 (5.048-18.773) |
|  | heat stroke | 6 | 9.301 | 3.725 | 3.726 (1.668-8.325) | intentional underdose | 8 | 4.98 | 2.361 | 2.362 (1.180-4.729) |
|  | ligament injury | 5 | 7.966 | 3.892 | 3.893 (1.613-9.393) | concussion | 7 | 5.632 | 2.652 | 2.653 (1.263-5.572) |
|  | heavy exposure to ultraviolet light | 4 | 65.378 | 24.911 | 24.915 (9.077-68.392) | facial bones fracture | 6 | 8.503 | 3.538 | 3.539 (1.588-7.891) |
|  | burns third degree | 4 | 4.953 | 3.551 | 3.551 (1.327-9.504) | traumatic lung injury | 5 | 13.846 | 5.432 | 5.435 (2.256-13.09) |
|  | asbestosis | 3 | 17.573 | 10.941 | 10.942 (3.476-34.449) | eye contusion | 4 | 13.439 | 6.502 | 6.504 (2.433-17.386) |
|  |  |  |  |  |  | foreign body in throat | 4 | 8.285 | 4.728 | 4.729 (1.771-12.632) |
|  |  |  |  |  |  | skin abrasion | 4 | 4.304 | 3.3 | 3.301 (1.237-8.811) |
|  |  |  |  |  |  | skin wound | 4 | 29.539 | 11.935 | 11.94 (4.455-32.000) |
|  |  |  |  |  |  | bone contusion | 3 | 26.822 | 15.377 | 15.382 (4.919-48.102) |
|  |  |  |  |  |  | transplantation complication | 3 | 33.078 | 18.489 | 18.495 (5.904-57.935) |
| Investigations | weight decreased | 1288 | 3959.129 | 4.876 | 5.086 (4.807-5.381) | weight decreased | 786 | 4494.225 | 7.575 | 8.147 (7.572-8.765) |
|  | oxygen saturation decreased | 344 | 2234.248 | 8.525 | 8.63 (7.750-9.610) | oxygen saturation decreased | 234 | 2944.874 | 14.734 | 15.069 (13.224-17.171) |
|  | hepatic enzyme increased | 134 | 68.131 | 2.023 | 2.028 (1.711-2.405) | hepatic enzyme increased | 194 | 1082.864 | 7.508 | 7.639 (6.623-8.810) |
|  | pulmonary function test decreased | 110 | 2757.402 | 29.085 | 29.209 (24.061-35.458) | blood pressure increased | 129 | 79.769 | 2.162 | 2.178 (1.830-2.592) |
|  | pulmonary function test abnormal | 85 | 4356.963 | 61.775 | 61.982 (49.317-77.899) | liver function test increased | 82 | 715.195 | 10.834 | 10.916 (8.774-13.582) |
|  | liver function test increased | 71 | 133.648 | 3.662 | 3.67 (2.904-4.637) | oxygen consumption increased | 66 | 5069.573 | 86.502 | 87.081 (67.688-112.028) |
|  | forced vital capacity decreased | 45 | 3729.341 | 109.738 | 109.933 (79.062-152.859) | pulmonary function test decreased | 45 | 1164.558 | 29.194 | 29.323 (21.79-39.461) |
|  | blood sodium decreased | 43 | 30.333 | 2.305 | 2.307 (1.709-3.115) | transaminases increased | 43 | 113.141 | 4.499 | 4.514 (3.343-6.094) |
|  | oxygen consumption increased | 37 | 562.131 | 18.288 | 18.314 (13.171-25.465） | gamma-glutamyltransferase increased | 38 | 94.884 | 4.364 | 4.377 (3.181-6.023) |
|  | international normalised ratio decreased | 26 | 70.787 | 4.68 | 4.684 (3.181-6.896) | blood potassium decreased | 36 | 56.165 | 3.327 | 3.335 (2.403-4.629) |
|  | total lung capacity decreased | 22 | 317.76 | 17.776 | 17.791 (11.606-27.271) | blood urine present | 30 | 62.39 | 3.928 | 3.937 (2.749-5.638) |
|  | lung diffusion test decreased | 19 | 1276.689 | 88.407 | 88.473 (53.850-145.356) | blood alkaline phosphatase increased | 27 | 23.898 | 2.551 | 2.555 (1.751-3.73) |
|  | carbon monoxide diffusing capacity decreased | 15 | 977.409 | 86.745 | 86.796 (49.688-151.618) | oxygen saturation abnormal | 27 | 884.166 | 37.225 | 37.325 (25.412-54.822) |
|  | oxygen saturation abnormal | 14 | 70.158 | 7.418 | 7.422 (4.374-12.593) | blood pressure abnormal | 20 | 20.099 | 2.724 | 2.728 (1.758-4.233) |
|  | red blood cell count increased | 9 | 14.715 | 3.662 | 3.663 (1.900-7.061) | catheterisation cardiac | 20 | 135.985 | 9.151 | 9.167 (5.9-14.243) |
|  | fev1/fvc ratio decreased | 6 | 268.766 | 63.917 | 63.932 (27.024-151.251) | heart rate irregular | 20 | 9.248 | 2.017 | 2.019 (1.301-3.132) |
|  | sputum abnormal | 6 | 32.857 | 8.706 | 8.707 (3.878-19.55) | blood sodium decreased | 18 | 14.147 | 2.46 | 2.463 (1.550-3.913) |
|  | investigation abnormal | 6 | 21.258 | 6.276 | 6.277 (2.803-14.061) | forced vital capacity decreased | 17 | 1305.746 | 90.731 | 90.887 (55.338-149.272) |
|  | gastric ph decreased | 6 | 17.374 | 5.458 | 5.459 (2.439-12.218) | pulmonary function test abnormal | 17 | 407.541 | 28.163 | 28.210 (17.418-45.690) |
|  | walking distance test abnormal | 4 | 24.334 | 10.314 | 10.315 (3.823-27.829) | hepatic enzyme abnormal | 15 | 73.461 | 7.197 | 7.206 (4.335-11.979) |
|  | blood bilirubin decreased | 4 | 16.484 | 7.602 | 7.603 (2.827-20.446) | oxygen saturation increased | 15 | 1851.401 | 153.773 | 154.007 (89.504-264.994) |
|  | mean cell haemoglobin increased | 4 | 5.281 | 3.672 | 3.672 (1.372-9.829) | blood magnesium decreased | 14 | 41.394 | 5.056 | 5.062 (2.993-8.561) |
|  | carbon dioxide increased | 4 | 4.116 | 3.238 | 3.239 (1.211-8.664) | blood test abnormal | 14 | 13.258 | 2.696 | 2.698 (1.596-4.561) |
|  | immature granulocyte count increased | 3 | 51.601 | 28.915 | 28.918 (8.963-93.304) | total lung capacity decreased | 12 | 239.822 | 24.315 | 24.343 (13.729-43.164) |
|  | liver function test decreased | 3 | 12.091 | 8.151 | 8.151 (2.599-25.564) | sars-cov-2 test positive | 11 | 10.724 | 2.772 | 2.774 (1.534-5.014) |
|  | vital functions abnormal | 3 | 5.203 | 4.618 | 4.618 (1.480-14.412) | blood iron decreased | 10 | 11.083 | 2.963 | 2.965 (1.593-5.516) |
|  |  |  |  |  |  | haemoglobin abnormal | 10 | 23.915 | 4.521 | 4.525 (2.431-8.424) |
|  |  |  |  |  |  | oxygen consumption | 9 | 755.791 | 105.894 | 105.99 (53.364-210.515) |
|  |  |  |  |  |  | respiratory rate increased | 8 | 5.309 | 2.42 | 2.421 (1.209-4.846) |
|  |  |  |  |  |  | oxygen consumption decreased | 7 | 142.691 | 26.356 | 26.374 (12.453-55.858) |
|  |  |  |  |  |  | heart rate abnormal | 6 | 5.363 | 2.828 | 2.829 (1.269-6.305) |
|  |  |  |  |  |  | blood electrolytes decreased | 5 | 34.166 | 10.63 | 10.635 (4.406-25.673) |
|  |  |  |  |  |  | carbon dioxide increased | 5 | 33.161 | 10.375 | 10.38 (4.300-25.053) |
|  |  |  |  |  |  | haemoglobin increased | 5 | 5.475 | 3.192 | 3.193 (1.327-7.683) |
|  |  |  |  |  |  | lung diffusion test decreased | 5 | 189.279 | 51.258 | 51.283 (20.888-125.908) |
|  |  |  |  |  |  | respiratory rate decreased | 5 | 6.633 | 3.515 | 3.516 (1.461-8.462) |
|  |  |  |  |  |  | walking distance test abnormal | 5 | 121.63 | 33.186 | 33.202 (13.625-80.908) |
|  |  |  |  |  |  | aspartate aminotransferase abnormal | 4 | 19.718 | 8.628 | 8.631 (3.226-23.097) |
|  |  |  |  |  |  | human chorionic gonadotropin increased | 4 | 25.956 | 10.73 | 10.734 (4.007-28.75) |
|  |  |  |  |  |  | alanine aminotransferase abnormal | 3 | 5.046 | 4.515 | 4.516 (1.453-14.039) |
|  |  |  |  |  |  | blood immunoglobulin g increased | 3 | 8.967 | 6.512 | 6.514 (2.093-20.272) |
|  |  |  |  |  |  | carbon monoxide diffusing capacity decreased | 3 | 71.358 | 37.881 | 37.892 (11.971-119.944) |
|  |  |  |  |  |  | catheterisation cardiac abnormal | 3 | 48.259 | 26.103 | 26.11 (8.301-82.128) |
|  |  |  |  |  |  | gamma-glutamyltransferase abnormal | 3 | 28.951 | 16.435 | 16.44 (5.254-51.440) |
|  |  |  |  |  |  | myocardial necrosis marker increased | 3 | 8.833 | 6.444 | 6.446 (2.071-20.061) |
|  |  |  |  |  |  | oxygen saturation | 3 | 70.472 | 37.424 | 37.435 (11.829-118.47) |
|  |  |  |  |  |  | pulmonary arterial pressure increased | 3 | 5.361 | 4.678 | 4.679 (1.505-14.548) |
|  |  |  |  |  |  | total lung capacity abnormal | 3 | 89.03 | 47.064 | 47.078 (14.800-149.757) |
| Metabolism and nutrition disorders | decreased appetite | 2016 | 15546.899 | 9.732 | 10.495 (10.023-10.990) | decreased appetite | 847 | 7104.907 | 10.314 | 11.194 (10.429-12.015) |
|  | feeding disorder | 71 | 201.622 | 4.711 | 4.721 (3.735-5.968) | dehydration | 227 | 635.439 | 4.596 | 4.681 (4.102-5.341) |
|  | hypophagia | 60 | 64.901 | 2.754 | 2.758 (2.139-3.557) | abnormal loss of weight | 54 | 1229.238 | 25.742 | 25.879 (19.741-33.926) |
|  | appetite disorder | 26 | 63.964 | 4.387 | 4.391 (2.983-6.464) | fluid retention | 52 | 44.496 | 2.481 | 2.489 (1.895-3.270) |
|  | food aversion | 18 | 198.685 | 14.067 | 14.076 (8.797-22.524) | hypophagia | 33 | 66.981 | 3.863 | 3.872 (2.750-5.453) |
|  | haemochromatosis | 5 | 12.561 | 5.124 | 5.125 (2.121-12.383) | feeding disorder | 26 | 64.246 | 4.38 | 4.389 (2.984-6.455) |
|  | cow's milk intolerance | 3 | 21.986 | 13.2 | 13.202 (4.180-41.693) | gout | 17 | 12.949 | 2.435 | 2.437 (1.514-3.924) |
|  |  |  |  |  |  | appetite disorder | 15 | 63.273 | 6.444 | 6.453 (3.882-10.725) |
|  |  |  |  |  |  | food aversion | 13 | 277.923 | 25.737 | 25.769 (14.858-44.695) |
|  |  |  |  |  |  | electrolyte imbalance | 10 | 7.242 | 2.459 | 2.460 (1.322-4.577) |
|  |  |  |  |  |  | malnutrition | 8 | 4.579 | 2.289 | 2.290 (1.144-4.584) |
|  |  |  |  |  |  | lactose intolerance | 6 | 19.567 | 5.883 | 5.886 (2.638-13.135) |
|  |  |  |  |  |  | diet refusal | 3 | 13.603 | 8.824 | 8.827 (2.833-27.506) |
|  |  |  |  |  |  | food refusal | 3 | 26.22 | 15.079 | 15.083 (4.824-47.160) |
|  |  |  |  |  |  | hypervolaemia | 3 | 4.24 | 4.093 | 4.093 (1.317-12.723) |
| Neoplasms (benign, malignant, and unspecified) | lung neoplasm | 15 | 12.241 | 2.524 | 2.525 (1.519-4.195) | malignant neoplasm progression | 279 | 1447.941 | 7.085 | 7.263 (6.445-8.184) |
|  | small cell lung cancer | 14 | 118.732 | 11.357 | 11.363 (6.680-19.33) | lung neoplasm malignant | 58 | 43.153 | 2.339 | 2.347 (1.813-3.039) |
|  | bronchial carcinoma | 4 | 6.288 | 4.038 | 4.038 (1.508-10.814) | metastases to central nervous system | 23 | 61.695 | 4.633 | 4.642 (3.080-6.995) |
|  | pleural neoplasm | 3 | 32.547 | 18.684 | 18.686 (5.872-59.457) | non-small cell lung cancer | 22 | 251.518 | 14.105 | 14.134 (9.276-21.537) |
|  |  |  |  |  |  | colon cancer | 18 | 15.306 | 2.542 | 2.545 (1.602-4.044) |
|  |  |  |  |  |  | metastases to liver | 17 | 14.275 | 2.535 | 2.537 (1.576-4.086) |
|  |  |  |  |  |  | small cell lung cancer | 9 | 129.592 | 18.489 | 18.505 (9.570-35.784) |
|  |  |  |  |  |  | lung adenocarcinoma | 8 | 33.999 | 6.834 | 6.839 (3.411-13.711) |
|  |  |  |  |  |  | lung neoplasm | 8 | 11.43 | 3.433 | 3.435 (1.715-6.878) |
|  |  |  |  |  |  | metastases to meninges | 7 | 42.149 | 9.071 | 9.077 (4.312-19.107) |
|  |  |  |  |  |  | adenocarcinoma | 6 | 19.196 | 5.806 | 5.809 (2.603-12.962) |
|  |  |  |  |  |  | bronchial carcinoma | 6 | 66.868 | 15.57 | 15.579 (6.955-34.894) |
|  |  |  |  |  |  | metastases to lymph nodes | 6 | 4.106 | 2.527 | 2.528 (1.134-5.635) |
|  |  |  |  |  |  | squamous cell carcinoma of lung | 6 | 106.784 | 23.802 | 23.816 (10.600-53.513) |
|  |  |  |  |  |  | malignant neoplasm of pleura | 5 | 395.033 | 110.149 | 110.205 (43.822-277.145) |
|  |  |  |  |  |  | bile duct cancer | 4 | 12.886 | 6.313 | 6.316 (2.363-16.881) |
|  |  |  |  |  |  | gastrointestinal neoplasm | 3 | 12.166 | 8.11 | 8.112 (2.604-25.269) |
|  |  |  |  |  |  | lung carcinoma cell type unspecified recurrent | 3 | 24.548 | 14.249 | 14.253 (4.560-44.544) |
|  |  |  |  |  |  | metastases to spleen | 3 | 32.427 | 18.165 | 18.17 (5.802-56.909) |
|  |  |  |  |  |  | phaeochromocytoma | 3 | 13.807 | 8.926 | 8.928 (2.865-27.823) |
|  |  |  |  |  |  | sarcoma | 3 | 6.978 | 5.507 | 5.509 (1.771-17.135) |
|  |  |  |  |  |  | pleural neoplasm | 3 | 90.411 | 47.788 | 47.802 (15.021-152.119) |
| Nervous system disorders | dizziness | 1303 | 1055.729 | 2.378 | 2.453 (2.320-2.594) | dysgeusia | 80 | 69.321 | 2.481 | 2.493 (2.000-3.108) |
|  | dysgeusia | 239 | 296.947 | 2.912 | 2.930 (2.579-3.330) | taste disorder | 69 | 865.692 | 14.853 | 14.951 (11.779-18.977) |
|  | ageusia | 130 | 516.622 | 5.896 | 5.922 (4.978-7.045) | ageusia | 58 | 272.664 | 6.675 | 6.709 (5.178-8.691) |
|  | hypersomnia | 88 | 108.362 | 2.918 | 2.925 (2.370-3.608) | transient ischaemic attack | 55 | 92.907 | 3.446 | 3.460 (2.653-4.512) |
|  | taste disorder | 72 | 295.102 | 6.063 | 6.078 (4.814-7.673) | cerebral haemorrhage | 43 | 39.796 | 2.574 | 2.581 (1.912-3.484) |
|  | anosmia | 26 | 24.799 | 2.644 | 2.646 (1.799-3.891) | hypersomnia | 40 | 64.505 | 3.379 | 3.389 (2.483-4.625) |
|  | parosmia | 21 | 21.333 | 2.739 | 2.74 (1.784-4.210) | cerebral infarction | 36 | 50.688 | 3.153 | 3.161 (2.277-4.387) |
|  | hyperaesthesia | 20 | 11.388 | 2.168 | 2.169 (1.398-3.367) | aphasia | 26 | 12.094 | 2.005 | 2.007 (1.365-2.951) |
|  | hypogeusia | 14 | 81.637 | 8.347 | 8.351 (4.918-14.178) | head discomfort | 23 | 39.861 | 3.568 | 3.574 (2.372-5.384) |
|  | hemiplegic migraine | 5 | 71.995 | 20.866 | 20.87 (8.494-51.278) | polyneuropathy | 20 | 61.25 | 5.08 | 5.089 (3.278-7.900) |
|  |  |  |  |  |  | ischaemic stroke | 18 | 9.818 | 2.141 | 2.143 (1.349-3.404) |
|  |  |  |  |  |  | dizziness postural | 11 | 15.204 | 3.282 | 3.285 (1.817-5.939) |
|  |  |  |  |  |  | cerebral disorder | 9 | 9.243 | 2.883 | 2.885 (1.499-5.551) |
|  |  |  |  |  |  | sinus headache | 8 | 9.007 | 3.044 | 3.046 (1.521-6.099) |
|  |  |  |  |  |  | tension headache | 6 | 10.566 | 3.987 | 3.989 (1.789-8.896) |
|  |  |  |  |  |  | carotid artery disease | 3 | 5.347 | 4.671 | 4.672 (1.503-14.526) |
| Respiratory, thoracic, and mediastinal | dyspnoea | 1493 | 1469.978 | 2.582 | 2.682 (2.545-2.826） | dyspnoea | 1130 | 3668.791 | 4.99 | 5.509 (5.177-5.862) |
|  | cough | 1012 | 2248.455 | 3.972 | 4.097 (3.846-4.364) | cough | 762 | 4396.804 | 7.631 | 8.189 (7.603-8.819) |
|  | productive cough | 307 | 2017.204 | 8.609 | 8.704 (7.768-9.752) | idiopathic pulmonary fibrosis | 736 | 384550.06 | 1061.361 | 1147.264 (1033.066-1274.085) |
|  | idiopathic pulmonary fibrosis | 247 | 16512.305 | 82.84 | 83.654 (72.911-95.979) | productive cough | 406 | 10789.152 | 29.323 | 30.544 (27.619-33.780) |
|  | rhinorrhoea | 196 | 303.076 | 3.26 | 3.278 (2.846-3.774) | dyspnoea exertional | 270 | 5535.119 | 22.967 | 23.588 (20.874-26.656) |
|  | lung disorder | 179 | 332.201 | 3.606 | 3.625 (3.127-4.201) | epistaxis | 221 | 1159.719 | 7.155 | 7.297 (6.383-8.342) |
|  | respiratory failure | 159 | 85.282 | 2.058 | 2.065 (1.766-2.414) | respiratory failure | 173 | 669.218 | 5.731 | 5.815 (5.002-6.762) |
|  | dyspnoea exertional | 156 | 510.205 | 5.14 | 5.166 (4.409-6.053) | interstitial lung disease | 145 | 906.934 | 8.221 | 8.329 (7.065-9.820) |
|  | pulmonary fibrosis | 135 | 810.714 | 8.049 | 8.087 (6.817-9.593) | pneumothorax | 122 | 2250.185 | 20.921 | 21.171 (17.678-25.355) |
|  | pneumothorax | 85 | 317.541 | 5.664 | 5.68 (4.584-7.038) | pulmonary embolism | 120 | 112.799 | 2.56 | 2.579 (2.154-3.089) |
|  | pulmonary hypertension | 81 | 128.911 | 3.332 | 3.339 (2.682-4.157) | haemoptysis | 101 | 724.139 | 9.198 | 9.283 (7.624-11.303) |
|  | haemoptysis | 76 | 79.209 | 2.7 | 2.705 (2.158-3.391) | pulmonary fibrosis | 92 | 1078.456 | 13.941 | 14.063 (11.437-17.292) |
|  | respiratory disorder | 73 | 53.373 | 2.322 | 2.326 (1.847-2.928) | lung disorder | 89 | 243.665 | 4.565 | 4.598 (3.730-5.668) |
|  | sneezing | 69 | 108.13 | 3.31 | 3.316 (2.616-4.204) | pulmonary hypertension | 76 | 454.817 | 7.991 | 8.046 (6.415-10.092) |
|  | acute respiratory failure | 52 | 75.539 | 3.192 | 3.196 (2.432-4.200) | hypoxia | 67 | 226.706 | 5.27 | 5.3 (4.165-6.743) |
|  | pulmonary thrombosis | 34 | 43.067 | 3.001 | 3.003 (2.143-4.209) | dysphonia | 65 | 83.692 | 2.984 | 2.997 (2.347-3.826) |
|  | sinus congestion | 30 | 23.56 | 2.424 | 2.426 (1.694-3.474) | rhinorrhoea | 64 | 67.224 | 2.708 | 2.719 (2.126-3.478) |
|  | upper-airway cough syndrome | 29 | 66.282 | 4.178 | 4.181 (2.900-6.030) | pleural effusion | 59 | 49.676 | 2.46 | 2.469 (1.911-3.190) |
|  | sputum increased | 27 | 467.678 | 20.898 | 20.92 (14.206-30.806) | chronic obstructive pulmonary disease | 48 | 33.642 | 2.291 | 2.297 (1.730-3.052) |
|  | emphysema | 26 | 20.211 | 2.423 | 2.424 (1.648-3.566) | wheezing | 45 | 29.181 | 2.226 | 2.232 (1.665-2.992) |
|  | pulmonary pain | 24 | 224.342 | 11.994 | 12.005 (7.998-18.020) | respiratory disorder | 44 | 78.728 | 3.571 | 3.583 (2.663-4.820） |
|  | sputum discoloured | 24 | 35.441 | 3.277 | 3.279 (2.194-4.901) | nasal congestion | 42 | 20.647 | 2.021 | 2.025 (1.495-2.743) |
|  | paranasal sinus hypersecretion | 22 | 78.106 | 5.629 | 5.634 (3.698-8.582) | acute respiratory failure | 39 | 160.787 | 6.111 | 6.131 (4.473-8.405) |
|  | rales | 22 | 55.119 | 4.464 | 4.467 (2.934-6.801) | pneumonitis | 35 | 69.53 | 3.809 | 3.819 (2.739-5.326) |
|  | painful respiration | 21 | 134.047 | 8.773 | 8.779 (5.697-13.53) | respiratory tract congestion | 34 | 150.533 | 6.457 | 6.476 (4.620-9.078) |
|  | lower respiratory tract congestion | 19 | 250.606 | 16.47 | 16.482 (10.416-26.079) | pulmonary arterial hypertension | 28 | 63.919 | 4.162 | 4.171 (2.876-6.048) |
|  | bronchiectasis | 13 | 10.033 | 2.485 | 2.485 (1.441-4.288) | respiration abnormal | 28 | 242.738 | 11.023 | 11.052 (7.612-16.047) |
|  | upper respiratory tract congestion | 11 | 17.378 | 3.534 | 3.535 (1.952-6.401) | sputum discoloured | 28 | 210.37 | 9.791 | 9.816 (6.762-14.249) |
|  | pharyngeal disorder | 9 | 8.632 | 2.8 | 2.801 (1.454-5.396) | pulmonary mass | 26 | 100.675 | 5.91 | 5.923 (4.026-8.713) |
|  | chronic respiratory failure | 7 | 29.508 | 6.962 | 6.964 (3.299-14.702) | pulmonary thrombosis | 24 | 81.434 | 5.406 | 5.416 (3.625-8.093) |
|  | nasal disorder | 7 | 4.153 | 2.354 | 2.354 (1.120-4.949) | pulmonary haemorrhage | 23 | 104.118 | 6.646 | 6.660 (4.418-10.04) |
|  | catarrh | 6 | 47.818 | 11.848 | 11.851 (5.262-26.69) | sneezing | 21 | 18.435 | 2.562 | 2.565 (1.671-3.938) |
|  | irregular breathing | 6 | 40.423 | 10.292 | 10.294 (4.578-23.149) | chronic respiratory failure | 20 | 914.233 | 52.559 | 52.664 (33.590-82.568) |
|  | pulmonary artery dilatation | 5 | 56.158 | 16.591 | 16.594 (6.784-40.586) | rales | 20 | 158.516 | 10.37 | 10.389 (6.685-16.145) |
|  | pharyngeal mass | 4 | 19.546 | 8.659 | 8.66 (3.216-23.319) | sinus disorder | 20 | 18.508 | 2.626 | 2.629 (1.695-4.079) |
|  | increased viscosity of bronchial secretion | 4 | 19.15 | 8.522 | 8.524 (3.166-22.947) | upper-airway cough syndrome | 20 | 102.681 | 7.346 | 7.359 (4.738-11.429) |
|  | restrictive pulmonary disease | 4 | 5.684 | 3.819 | 3.819 (1.427-10.224) | pneumomediastinum | 17 | 371.146 | 25.771 | 25.814 (15.947-41.787) |
|  | pulmonary artery aneurysm | 3 | 33.102 | 18.975 | 18.978 (5.962-60.41) | pulmonary pain | 17 | 306.448 | 21.545 | 21.580 (13.344-34.899) |
|  | paranasal sinus hyposecretion | 3 | 25.826 | 15.18 | 15.182 (4.794-48.077) | sputum increased | 16 | 422.681 | 31.023 | 31.072 (18.890-51.111) |
|  | ventilation perfusion mismatch | 3 | 22.852 | 13.645 | 13.647 (4.319-43.125) | dry throat | 14 | 22.143 | 3.47 | 3.474 (2.055-5.873) |
|  | idiopathic pneumonia syndrome | 3 | 21.44 | 12.919 | 12.921 (4.093-40.79) | aphonia | 13 | 10.265 | 2.504 | 2.506 (1.453-4.320) |
|  | nasal crusting | 3 | 9.634 | 6.9 | 6.901 (2.204-21.605) | pulmonary alveolar haemorrhage | 12 | 41.943 | 5.723 | 5.729 (3.247-10.107) |
|  | allergic sinusitis | 3 | 4.528 | 4.261 | 4.262 (1.366-13.293) | aspiration | 11 | 8.912 | 2.557 | 2.559 (1.416-4.626) |
|  |  |  |  |  |  | emphysema | 11 | 9.377 | 2.613 | 2.615 (1.446-4.726) |
|  |  |  |  |  |  | paranasal sinus hypersecretion | 11 | 51.842 | 7.15 | 7.157 (3.954-12.953) |
|  |  |  |  |  |  | cough decreased | 9 | 1154.185 | 169.43 | 169.584 (83.790-343.227) |
|  |  |  |  |  |  | bronchiectasis | 8 | 14.43 | 3.902 | 3.904 (1.949-7.819) |
|  |  |  |  |  |  | nasal dryness | 7 | 6.254 | 2.775 | 2.776 (1.322-5.831) |
|  |  |  |  |  |  | hypersensitivity pneumonitis | 5 | 71.734 | 20.223 | 20.233 (8.348-49.037) |
|  |  |  |  |  |  | increased viscosity of bronchial secretion | 5 | 99.466 | 27.392 | 27.405 (11.273-66.620) |
|  |  |  |  |  |  | painful respiration | 5 | 13.168 | 5.256 | 5.258 (2.183-12.664) |
|  |  |  |  |  |  | pneumothorax spontaneous | 5 | 77.678 | 21.752 | 21.763 (8.974-52.779) |
|  |  |  |  |  |  | respiratory symptom | 4 | 11.75 | 5.925 | 5.927 (2.218-15.840) |
|  |  |  |  |  |  | respiratory tract haemorrhage | 4 | 42.691 | 16.37 | 16.376 (6.098-43.981) |
|  |  |  |  |  |  | acute lung injury | 3 | 4.922 | 4.45 | 4.451 (1.432-13.838) |
|  |  |  |  |  |  | allergic cough | 3 | 22.697 | 13.331 | 13.335 (4.269-41.656) |
|  |  |  |  |  |  | lung cyst | 3 | 33.752 | 18.826 | 18.831 (6.010-58.999) |
|  |  |  |  |  |  | lung diffusion disorder | 3 | 132.994 | 70.596 | 70.617 (21.923-227.463) |
|  |  |  |  |  |  | pulmonary cavitation | 3 | 15.26 | 9.647 | 9.649 (3.095-30.082) |
|  |  |  |  |  |  | sputum retention | 3 | 21.275 | 12.627 | 12.63 (4.045-39.439) |
| Social circumstances | walking aid user | 27 | 73.72 | 4.683 | 4.687 (3.207-6.851) | bedridden | 11 | 4.602 | 2.009 | 2.010 (1.112-3.633) |
|  | dependence on oxygen therapy | 13 | 487.867 | 47.41 | 47.434 (26.700-84.271) | dependence on oxygen therapy | 3 | 47.404 | 25.671 | 25.679 (8.166-80.752) |
| Surgical and medical procedures | lung transplant | 45 | 342.197 | 9.884 | 9.900 (7.364-13.310) | lung transplant | 100 | 5243.523 | 57.908 | 58.494 (47.777-71.615) |
|  | oxygen therapy | 10 | 59.745 | 8.743 | 8.746 (4.674-16.366)) | stent placement | 18 | 44.719 | 4.459 | 4.465 (2.809-7.097) |
|  | sinus operation | 6 | 5.379 | 2.837 | 2.838 (1.271-6.335) | cardiac operation | 12 | 14.507 | 3.05 | 3.052 (1.731-5.381) |
|  | oesophageal operation | 3 | 18.374 | 11.35 | 11.351 (3.603-35.757) | cardiac pacemaker insertion | 11 | 21.012 | 3.919 | 3.923 (2.169-7.093) |
|  |  |  |  |  |  | oxygen therapy | 10 | 179.387 | 22.363 | 22.385 (11.960-42.895) |
|  |  |  |  |  |  | spinal operation | 9 | 7.056 | 2.561 | 2.562 (1.332-4.930) |
|  |  |  |  |  |  | colectomy | 7 | 13.901 | 4.189 | 4.192 (1.995-8.808) |
|  |  |  |  |  |  | orthopaedic procedure | 7 | 209.671 | 38.146 | 38.173 (17.949-81.184) |
|  |  |  |  |  |  | rehabilitation therapy | 7 | 19.395 | 5.159 | 5.162 (2.455-10.850) |
|  |  |  |  |  |  | cardiac ablation | 6 | 35.4 | 9.136 | 9.141 (4.091-20.424) |
|  |  |  |  |  |  | gallbladder operation | 6 | 8.394 | 3.514 | 3.515 (1.577-7.837) |
|  |  |  |  |  |  | dental operation | 5 | 13.148 | 5.251 | 5.253 (2.181-12.651) |
|  |  |  |  |  |  | eye operation | 5 | 5.996 | 3.338 | 3.339 (1.388-8.035) |
|  |  |  |  |  |  | lung operation | 5 | 49.695 | 14.583 | 14.59 (6.034-35.278) |
|  |  |  |  |  |  | sinus operation | 5 | 16.196 | 6.041 | 6.043 (2.508-14.560) |
|  |  |  |  |  |  | transplant | 5 | 8.833 | 4.112 | 4.114 (1.709-9.903) |
|  |  |  |  |  |  | lung lobectomy | 4 | 73.579 | 26.894 | 26.904 (9.969-72.612) |
|  |  |  |  |  |  | cholelithotomy | 3 | 52.995 | 28.497 | 28.506 (9.051-89.779) |
|  |  |  |  |  |  | pulmonary resection | 3 | 91.833 | 48.535 | 48.549 (15.250-154.558) |
| Skin and subcutaneous tissue | rash | 1322 | 1851.033 | 3.061 | 3.176 (3.004-3.357) | photosensitivity reaction | 24 | 35.839 | 3.287 | 3.292 (2.204-4.917) |
|  | pruritus | 680 | 371.512 | 2.057 | 2.087 (1.933-2.252) | skin lesion | 21 | 10.225 | 2.048 | 2.05 (1.335-3.147) |
|  | photosensitivity reaction | 471 | 10956.503 | 26.801 | 27.295 (24.844-29.988) | skin reaction | 19 | 27.31 | 3.25 | 3.254 (2.073-5.107) |
|  | dry skin | 155 | 122.859 | 2.385 | 2.393 (2.043-2.804) | rash papular | 14 | 6.484 | 2.048 | 2.049 (1.213-3.463) |
|  | rash pruritic | 128 | 116.946 | 2.535 | 2.543 (2.136-3.027) | skin haemorrhage | 9 | 5.262 | 2.283 | 2.285 (1.187-4.395) |
|  | skin exfoliation | 122 | 96.864 | 2.39 | 2.397 (2.005-2.865) | pemphigus | 8 | 22.342 | 5.104 | 5.107 (2.549-10.232) |
|  | rash erythematous | 83 | 50.957 | 2.169 | 2.173 (1.751-2.697) | blood blister | 6 | 14.983 | 4.927 | 4.929 (2.210-10.995) |
|  | skin reaction | 51 | 84.637 | 3.428 | 3.433 (2.605-4.524) | dermatitis acneiform | 6 | 5.038 | 2.751 | 2.752 (1.235-6.135) |
|  | solar dermatitis | 10 | 380.258 | 49.367 | 49.386 (25.612-95.231) | dermatitis exfoliative generalised | 5 | 11.202 | 4.741 | 4.743 (1.970-11.420) |
|  | dermatitis exfoliative generalised | 7 | 5.346 | 2.6 | 2.600 (1.236-5.468) | dermatomyositis | 5 | 12.105 | 4.978 | 4.98 (2.068-11.993) |
|  | subcutaneous emphysema | 4 | 6.288 | 4.038 | 4.038 (1.508-10.814) | solar lentigo | 5 | 65.237 | 18.556 | 18.565 (7.665-44.963) |
|  |  |  |  |  |  | subcutaneous emphysema | 5 | 43.254 | 12.943 | 12.949 (5.359-31.287) |
|  |  |  |  |  |  | perivascular dermatitis | 3 | 30.797 | 17.353 | 17.358 (5.545-54.341) |
| Vascular disorders | aortic aneurysm | 34 | 52.545 | 3.325 | 3.328 (2.374-4.665) | hypertension | 176 | 99.401 | 2.088 | 2.108 (1.816-2.448) |
|  | arterial occlusive disease | 20 | 12.315 | 2.231 | 2.232 (1.438-3.464) | internal haemorrhage | 45 | 237.84 | 7.323 | 7.352 (5.480-9.863) |
|  | aortic arteriosclerosis | 8 | 14.371 | 3.906 | 3.907 (1.947-7.840) | cyanosis | 18 | 17.275 | 2.68 | 2.683 (1.689-4.262) |
|  |  |  |  |  |  | blood pressure fluctuation | 16 | 11.983 | 2.424 | 2.427 (1.485-3.965) |
|  |  |  |  |  |  | aortic aneurysm | 12 | 13.884 | 2.985 | 2.988 (1.695-5.267) |
|  |  |  |  |  |  | peripheral ischaemia | 8 | 19.335 | 4.651 | 4.654 (2.323-9.323) |
|  |  |  |  |  |  | aneurysm | 7 | 7.438 | 3.004 | 3.005 (1.431-6.312) |
|  |  |  |  |  |  | aortic arteriosclerosis | 7 | 40.165 | 8.732 | 8.738 (4.152-18.391) |
|  |  |  |  |  |  | venous thrombosis | 7 | 13.996 | 4.207 | 4.209 (2.003-8.844) |
|  |  |  |  |  |  | peripheral arterial occlusive disease | 5 | 5.859 | 3.3 | 3.301 (1.237-8.811) |
|  |  |  |  |  |  | jugular vein thrombosis | 4 | 12.886 | 6.313 | 6.316 (2.363-16.881) |
|  |  |  |  |  |  | peripheral artery thrombosis | 4 | 18.647 | 8.267 | 8.27 (3.091-22.125) |
|  |  |  |  |  |  | venous thrombosis limb | 4 | 7.654 | 4.507 | 4.508 (1.688-12.04) |
| Ear and labyrinth disorders | excessive cerumen production | 3 | 9.188 | 6.673 | 6.673 (2.132-20.866) | motion sickness | 7 | 41.299 | 8.926 | 8.932 (4.243-18.800) |
|  |  |  |  |  |  | meniere's disease | 3 | 6.364 | 5.194 | 5.196 (1.671-16.158) |
| Eye disorders | amblyopia | 5 | 20.391 | 7.177 | 7.179 (2.965-17.383) | retinal artery occlusion | 5 | 9.63 | 4.325 | 4.327 (1.797-10.417) |
|  | conjunctival discolouration | 4 | 90.824 | 34.452 | 34.457 (12.414-95.645) |  |  |  |  |  |
| Immune system disorders | seasonal allergy | 28 | 24.653 | 2.548 | 2.549 (1.758-3.697) | seasonal allergy | 34 | 197.392 | 7.923 | 7.947 (5.668-11.143) |
|  | smoke sensitivity | 5 | 73.605 | 21.306 | 21.31 (8.669-52.382) | multiple allergies | 22 | 111.657 | 7.252 | 7.266 (4.775-11.057) |
| Musculoskeletal and connective tissue disorders | musculoskeletal chest pain | 32 | 20.572 | 2.237 | 2.239 (1.581-3.170) | musculoskeletal chest pain | 30 | 101.691 | 5.363 | 5.376 (3.753-7.701) |
|  | limb mass | 12 | 58.204 | 7.316 | 7.319 (4.135-12.955) | scleroderma | 25 | 283.87 | 13.947 | 13.98 (9.417-20.754) |
|  | clubbing | 9 | 145.796 | 21.182 | 21.189 (10.839-41.422) | myopathy | 12 | 13.337 | 2.928 | 2.931 (1.662-5.166) |
|  | bursa disorder | 6 | 80.463 | 18.828 | 18.833 (8.306-42.702) | systemic scleroderma | 11 | 529.066 | 57.814 | 57.878 (31.525-106.261) |
|  | groin infection | 3 | 8.014 | 6.072 | 6.073 (1.942-18.991) | sjogren's syndrome | 7 | 13.514 | 4.12 | 4.123 (1.962-8.663) |
|  | bone infarction | 3 | 9.634 | 6.9 | 6.901 (2.204-21.605) | clubbing | 4 | 63.38 | 23.399 | 23.408 (8.687-63.073) |
|  |  |  |  |  |  | lumbar spinal stenosis | 4 | 6.185 | 3.986 | 3.987 (1.493-10.646) |
| Psychiatric disorders | insomnia | 640 | 368.859 | 2.097 | 2.126 (1.965-2.300) | eating disorder | 39 | 97.559 | 4.366 | 4.38 (3.196-6.002) |
|  | eating disorder | 64 | 72.325 | 2.809 | 2.814 (2.200-3.599) | near death experience | 6 | 19.04 | 5.774 | 5.777 (2.589-12.89) |
|  |  |  |  |  |  | laziness | 3 | 4.616 | 4.29 | 4.291 (1.381-13.339) |
| Renal and urinary disorders | chromaturia | 80 | 125.973 | 3.313 | 3.320 (2.664-4.139) | chromaturia | 50 | 168.516 | 5.28 | 5.302 (4.013-7.005) |
|  | urine abnormality | 11 | 13.184 | 3.063 | 3.063 (1.693-5.545) | haematuria | 35 | 25.335 | 2.333 | 2.338 (1.677-3.260) |
|  |  |  |  |  |  | nephrotic syndrome | 11 | 19.15 | 3.717 | 3.72 (2.057-6.727) |
|  |  |  |  |  |  | urinary bladder haemorrhage | 10 | 86.812 | 11.739 | 11.75 (6.298-21.922) |
|  |  |  |  |  |  | renal pain | 9 | 4.118 | 2.097 | 2.098 (1.091-4.036) |
|  |  |  |  |  |  | urine odour abnormal | 9 | 14.296 | 3.592 | 3.595 (1.868-6.919) |
|  |  |  |  |  |  | haemorrhage urinary tract | 5 | 10.628 | 4.59 | 4.591 (1.907-11.055) |
|  |  |  |  |  |  | nephritis | 4 | 4.104 | 3.226 | 3.226 (1.209-8.612) |
|  |  |  |  |  |  | calculus bladder | 3 | 8.471 | 6.263 | 6.264 (2.013-19.493) |
|  |  |  |  |  |  | glomerulonephritis rapidly progressive | 3 | 8.886 | 6.471 | 6.473 (2.080-20.145) |
| Respiratory failure |  |  |  |  |  | cow's milk intolerance | 3 | 63.338 | 33.763 | 33.773 (10.693-106.671) |
| Reproductive system and breast disorders | penile discharge | 3 | 11.774 | 7.99 | 7.991 (2.548-25.054) | testicular disorder | 3 | 8.154 | 6.103 | 6.104 (1.962-18.933) |
|  | total | 38025 |  |  |  |  | 25658 |  |  |  |
